# Supplementary material for: Adaptive landscapes unveil the complex evolutionary path from sprawling to upright forelimb function and posture in mammals
Source: PLoS Biol. 2025 Jun 24;23(6):e3003188. doi: 10.1371/journal.pbio.3003188 (PMC12186895; doi:10.1371/journal.pbio.3003188)
Supplement: S1 Fig — Taxa are color-coded by group. The data underlying this figure can be found in S1 Table and S1 Data. (PDF) [file pbio.3003188.s009.pdf]

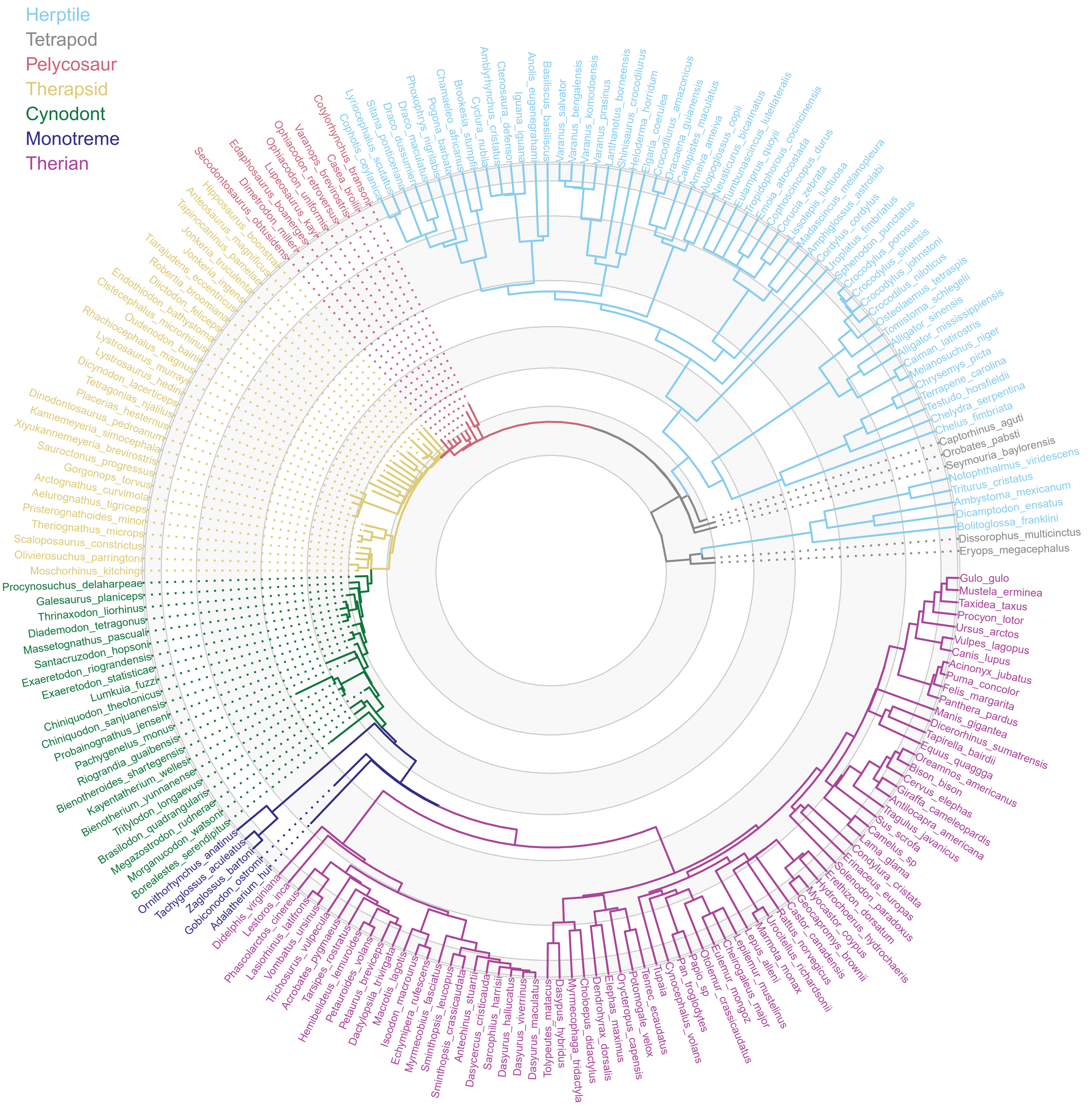

Herptile
Tetrapod
Pelycosaur
Therapsid
Cynodont
Monotreme
Therian
Lyriocephalus_scutatus
Cophotis_ceylanica
Sitana_ponticeriana
Draco_dussumieri
Draco_maculatus
Phoxophrys_nigrilabis
Pogona_barbata
Chamaeleo_africanus
Brookesia_stumpffi
Cyclura_nubila
Amblyrhynchus_cristatus
Ctenosaura_defensor
Iguana_iguana
Anolis_eugenegrahami
Basiliscus_basiliscus
Varanus_salvator
Varanus_bengalensis
Varanus_komodoensis
Varanus_prasinus
Lanthanotus_borneensis
Shinisaurus_crocodilurus
Heloderma_horridum
Elgaria_coerulea
Crocodilurus_amazonicus
Dracaena_guianensis
Callopistes_maculatus
Ameiva_ameiva
Alopoglossus_copii
Neusticurus_bicarinatus
Tumbunascincus_luteilateralis
Eulamprus_quoyii
Tropidophorus_cocincinensis
Emoia_atrocostata
Cophoscincopus_durus
Corucia_zebrata
Lissolepis_luctuosa
Madascincus_melanopleura
Amphiglossus_astrolabi
Cordylus_cordylus
Uroplatus_fimbriatus
Sphenodon_punctatus
Crocodylus_porosus
Crocodylus_sinensis
Crocodylus_johnstoni
Crocodylus_niloticus
Osteolaemus_tetraspis
Tomistoma_schlegelii
Alligator_sinensis
Alligator_mississippiensis
Caiman_latirostris
Melanosuchus_niger
Chrysemys_picta
Terrapene_carolina
Testudo_horsfieldii
Chelydra_serpentina
Chelus_fimbriata
Captorhinus_aguti
Orobates_pabsti
Seymouria_baylorensis
Notophthalmus_viridescens
Triturus_cristatus
Ambystoma_mexicanum
Dicamptodon_ensatus
Bolitoglossa_franklini
Dissorophus_multicinctus
Eryops_megacephalus
Gulo_gulo
Mustela_erminea
Taxidea_taxus
Procyon_lotor
Ursus_arctos
Vulpes_lagopus
Canis_lupus
Acinonyx_jubatus
Puma_concolor
Felis_margarita
Panthera_pardus
Manis_gigantea
Dicerorhinus_sumatrensis
Tapirella_bairdii
Equus_quaggga
Oreamnos_americanus
Bison_bison
Cervus_elephas
Giraffa_cameleopardis
Antilocapra_americana
Tragulus_javanicus
Sus_scrofa
Camelus_sp
Lama_glama
Condylura_cristata
Erinaceus_europas
Solenodon_paradoxus
Erethizon_dorsatum
Hydrochoerus_hydrochaeris
Myocastor_coypus
Geocapromys_brownii
Castor_canadensis
Rattus_norvegicus
Urocitellus_richardsonii
Marmota_monax
Lepus_alleni
Lepilemur_mustelinus
Cheirogaleus_major
Eulemur_mongoz
Otolemur_crassicaudatus
Papio_sp
Pan_troglodytes
Cynocephalus_volans
Tupaia
Tenrec_ecaudatus
Potamogale_velox
Orycteropus_capensis
Elephas_maximus
Dendrohyrax_dorsalis
Choloepus_didactylus
Myrmecophaga_tridactyla
Dasypus_hybridus
Tolypeutes_matacus
Dasyurus_maculatus
Dasyurus_viverrinus
Dasyurus_hallucatus
Sarcophilus_harrisii
Dasycercus_cristicauda
Antechinus_stuartii
Sminthopsis_crassicaudata
Sminthopsis_leucopus
Myrmecobius_fasciatus
Echymipera_rufescens
Isoodon_macrourus
Macrotis_lagotis
Dactylopsila_trivirgata
Petaurus_breviceps
Petauroides_volans
Hemibelideus_lemuroides
Tarsipes_rostratus
Acrobates_pygmaeus
Trichosurus_vulpecula
Vombatus_ursinus
Lasiorhinus_latifrons
Phascolarctos_cinereus
Lestoros_inca
Didelphis_virginiana
Adalatherium_hui
Gobiconodon_ostromi
Zaglossus_bartoni
Tachyglossus_aculeatus
Ornithorhynchus_anatinus
Borealestes_serendipitus
Morganucodon_watsoni
Megazostrodon_rudnerae
Brasilodon_quadrangularis
Tritylodon_longaevus
Bienotherium_yunnanense
Kayentatherium_wellesi
Bienotheroides_shartegensis
Riograndia_guaibensis
Pachygenelus_monus
Probainognathus_jenseni
Chiniquodon_sanjuanensis
Chiniquodon_theotonicus
Lumkuia_fuzzi
Exaeretodon_statisticae
Exaeretodon_riograndensis
Santacruzodon_hopsoni
Massetognathus_pascuali
Diademodon_tetragonus
Thrinaxodon_liorhinus
Galesaurus_planiceps
Procynosuchus_delaharpeae
Moschorhinus_kitchingi
Olivierosuchus_parringtoni
Scaloposaurus_constrictus
Theriognathus_microps
Pristerognathoides_minor
Aelurognathus_tigriceps
Arctognathus_curvimola
Gorgonops_torvus
Sauroctonus_progressus
Xiyukannemeyeria_brevirostris
Kannemeyeria_simocephalus
Dinodontosaurus_pedroanum
Placerias_hesternus
Tetragonias_njalilus
Dicynodon_lacerticeps
Lystrosaurus_hedini
Lystrosaurus_murrayi
Rhachiocephalus_magnus
Oudenodon_bainii
Cistecephalus_microrhinus
Endothiodon_bathystoma
Diictodon_feliceps
Robertia_broomiana
Tiarajudens_eccentricus
Jonkeria_ingens
Jonkeria_truculenta
Tapinocaninus_pamelae
Anteosaurus_magnificus
Hipposaurus_boonstrai
Secodontosaurus_obtusidens
Dimetrodon_milleri
Edaphosaurus_boanerges
Lupeosaurus_kayi
Ophiacodon_uniformis
Ophiacodon_retroversus
Varanops_brevirostris
Casea_broilii
Cotylorhynchus_bransoni
