# Supplementary figures and images for: Adaptive landscapes unveil the complex evolutionary path from sprawling to upright forelimb function and posture in mammals
Source: PLoS Biol. 2025 Jun 24;23(6):e3003188. doi: 10.1371/journal.pbio.3003188 (PMC12186895; doi:10.1371/journal.pbio.3003188)

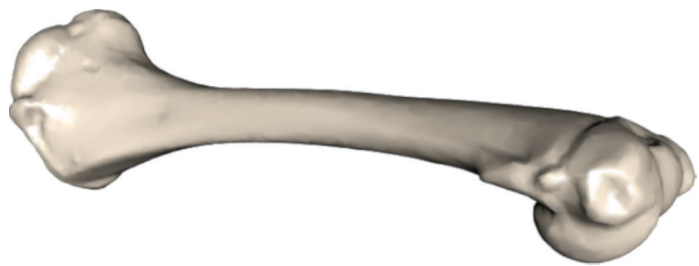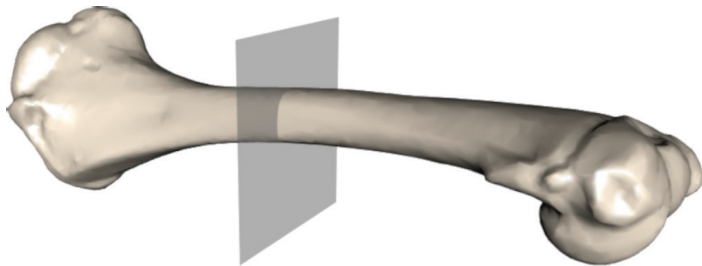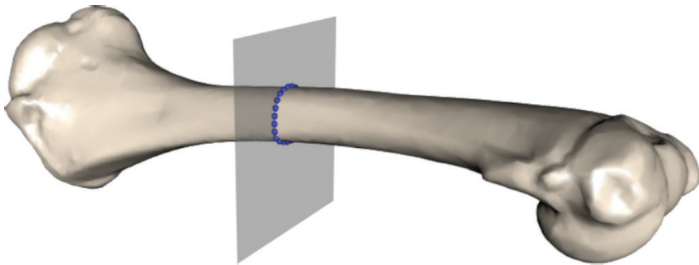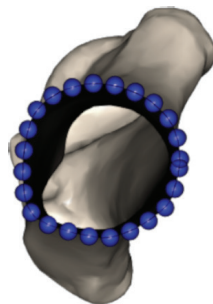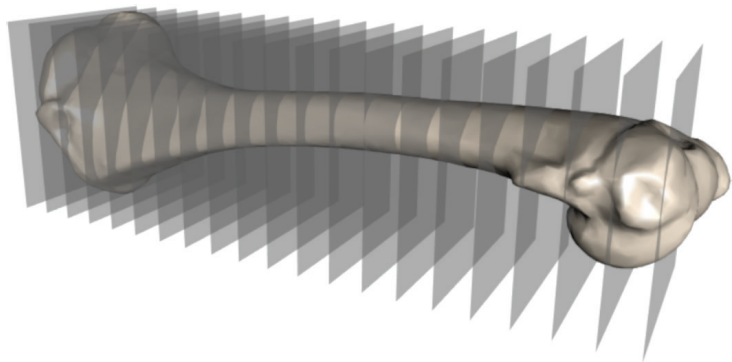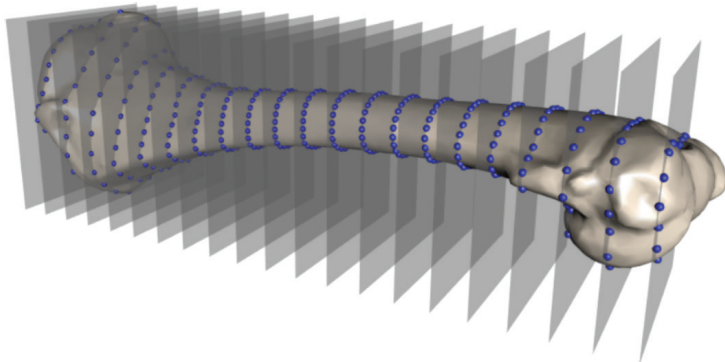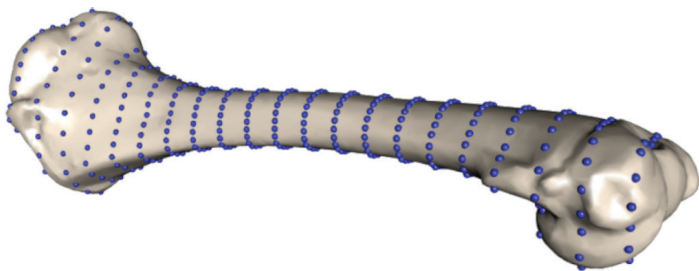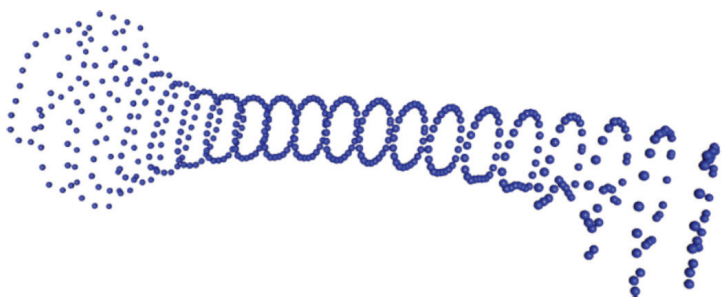

Supplement: S2 Fig — The top panels show a single plane slicing the mesh, and landmarks are placed around the contour resulting from the mesh-plane intersection. This is then repeated at intervals along the length of the bone to place landmarks across the entire surface. (PDF) [file pbio.3003188.s010.pdf]

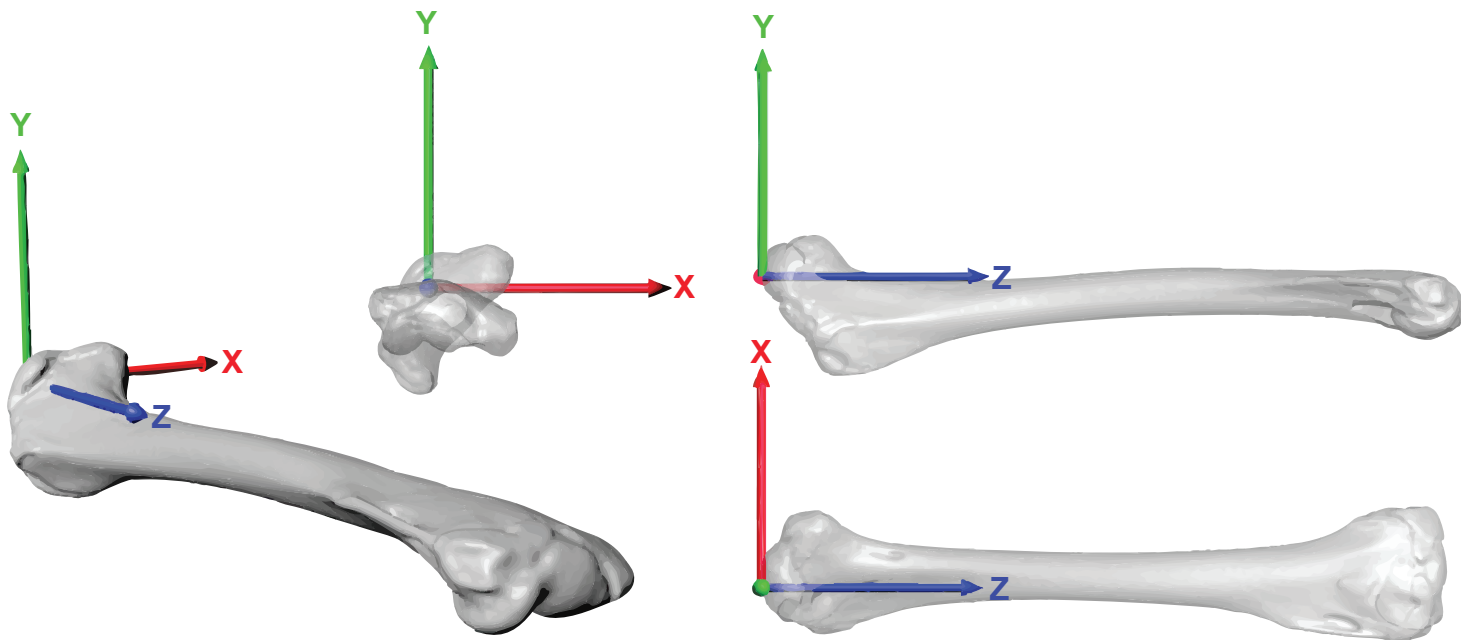

Humerus Length

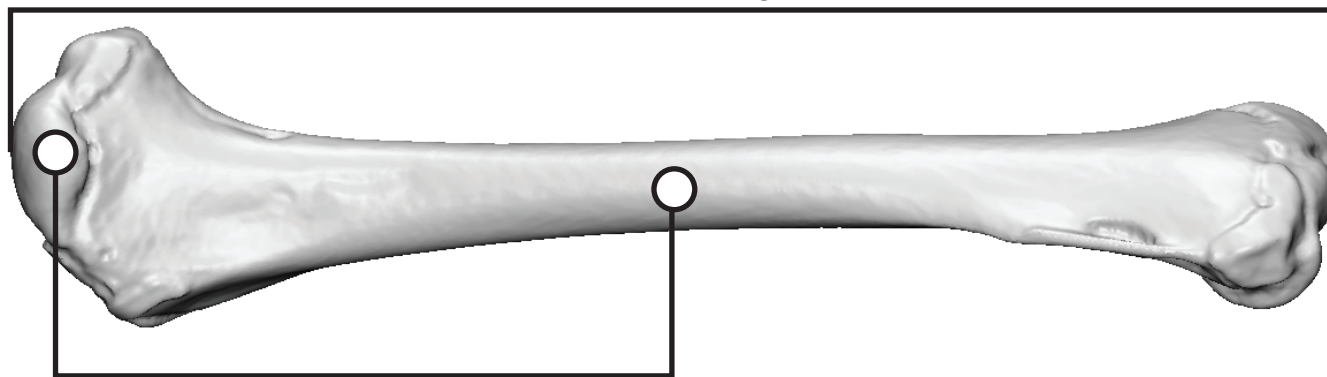

Radius of Gyration  
(Distance to Center of Mass)

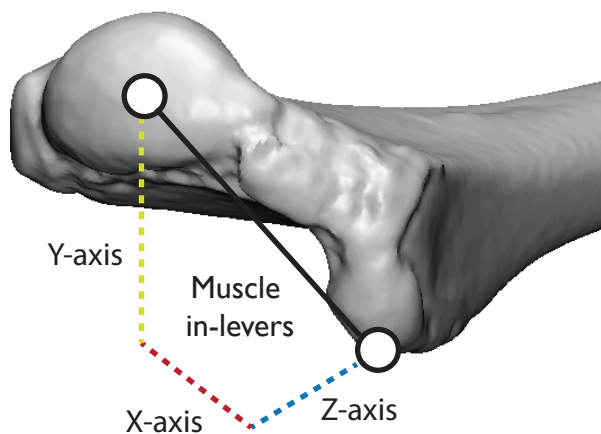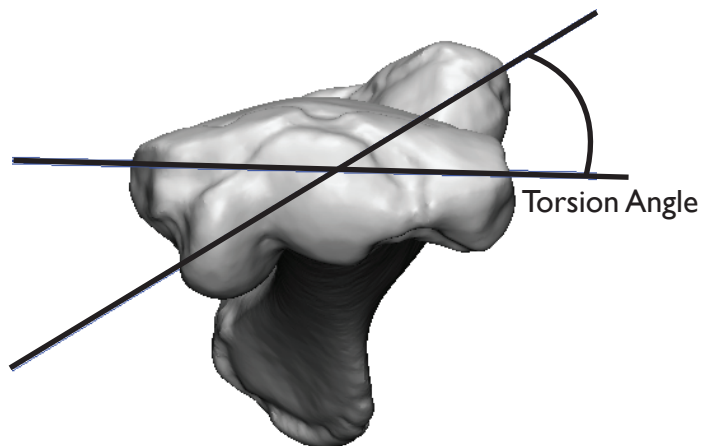

Supplement: S4 Fig — Prior to measurement, all humeri were aligned to a common coordinate system. All linear measurements were taken in mm, angular measurements were taken in degrees. Humerus length, distance from the center of mass to the center of rotation, in-lever for the muscles attaching to the deltopectoral crest (broken down in x, y, and z components) and torsion angle between the proximal and distal ends of the humerus. (PDF) [file pbio.3003188.s012.pdf]

Jenkins Tree

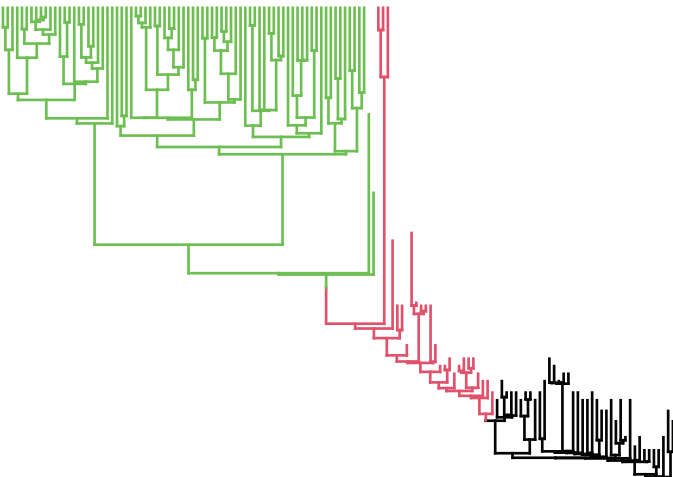

Romer Tree

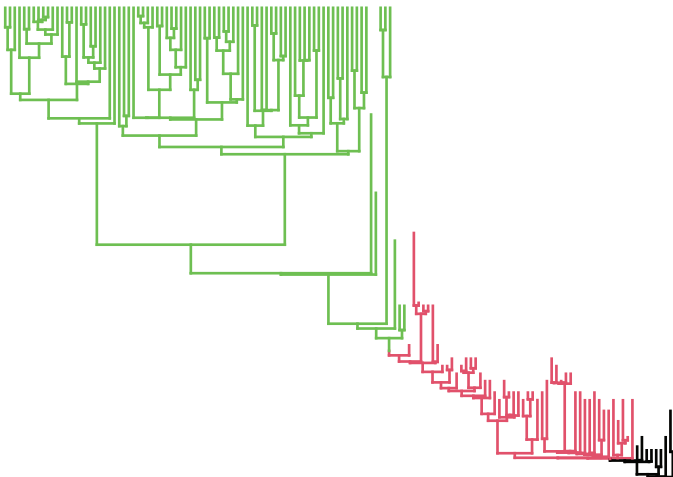

Kemp Tree

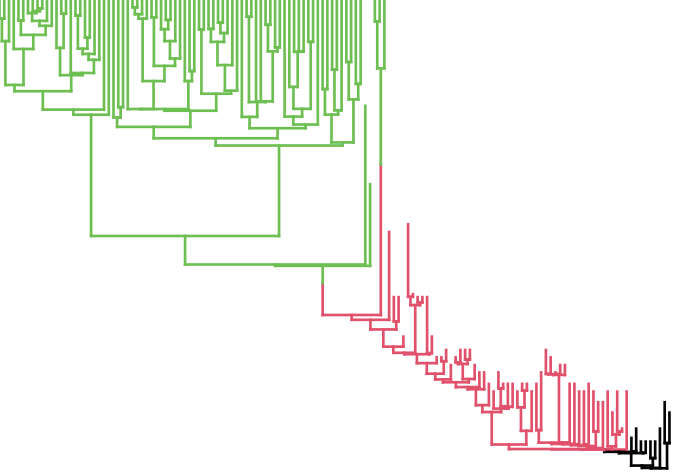

Grade Tree

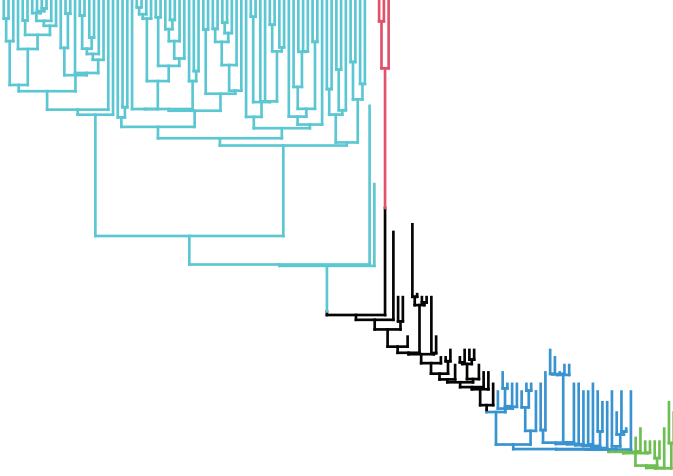

SubClade Tree

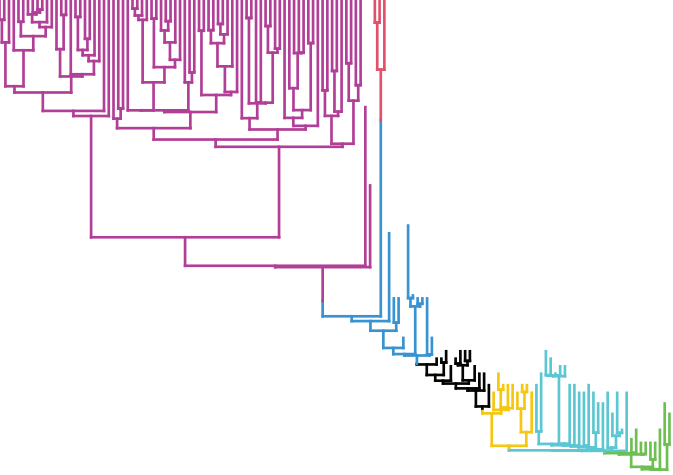

Supplement: S5 Fig — These are the hypotheses of synapsid forelimb transformation proposed by Jenkins, Romer, and Kemp. We also tested each major synapsid grade as its own regime, as well as adding sub-regimes for specific groups. The data underlying this figure can be found in S1 Table and S1 Data. (PDF) [file pbio.3003188.s013.pdf]

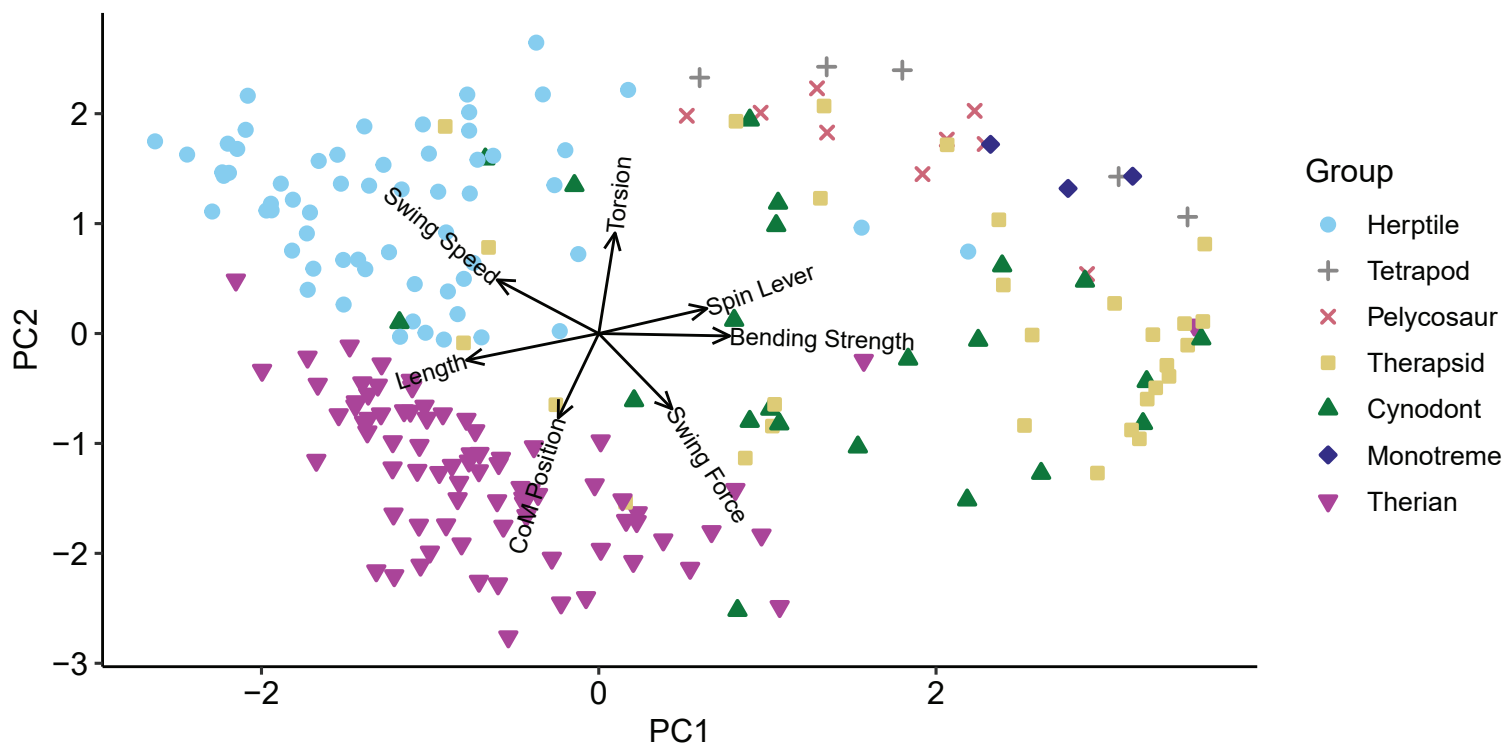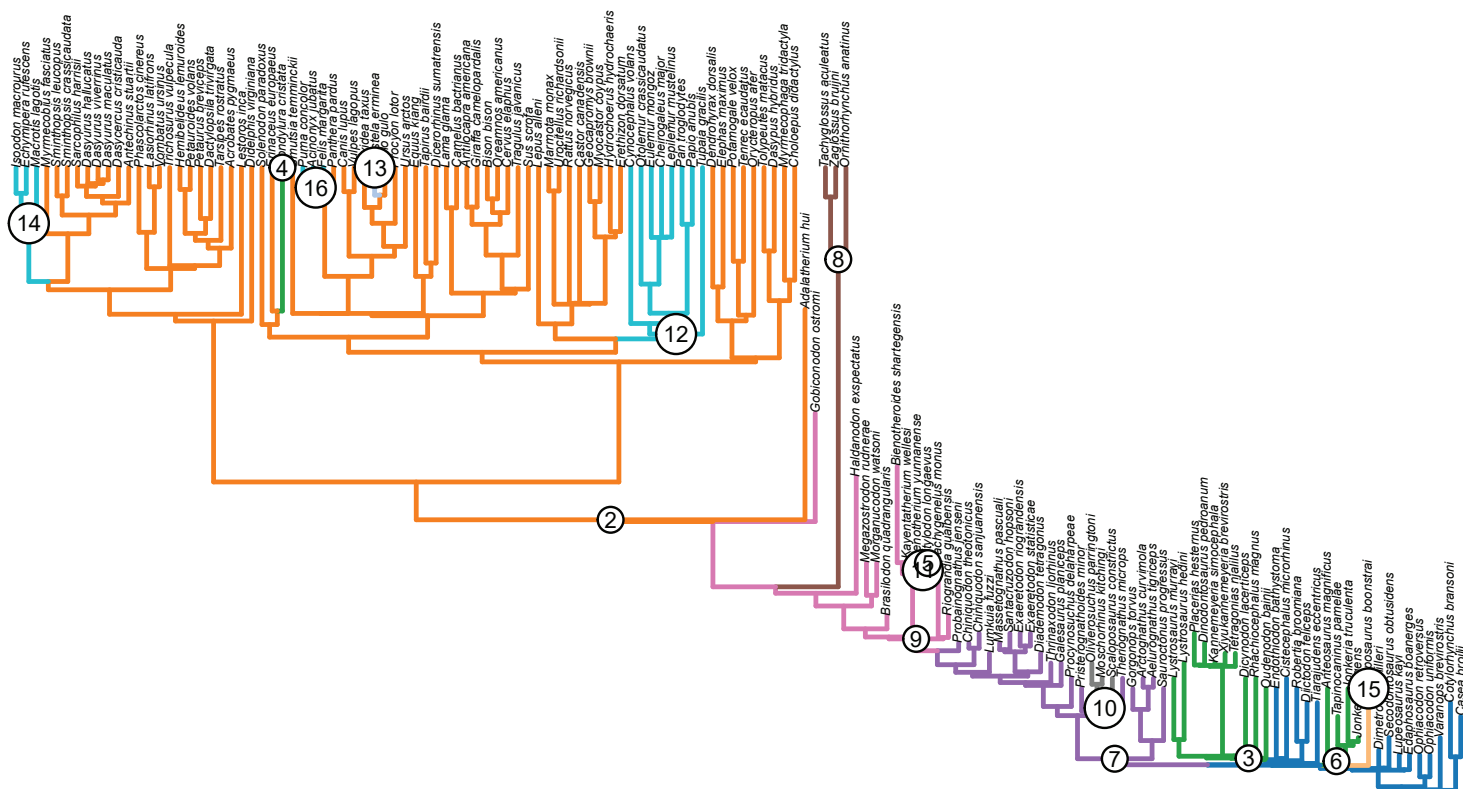

Supplement: S6 Fig — SURFACE analysis indicates shifts in the multi-variate evolutionary regime of adaptive landscape trait weights across synapsid evolution. Some convergent regimes were detected, and are color-coded the same. The data underlying this figure can be found in S1 Table and S1 Data. (PDF) [file pbio.3003188.s014.pdf]

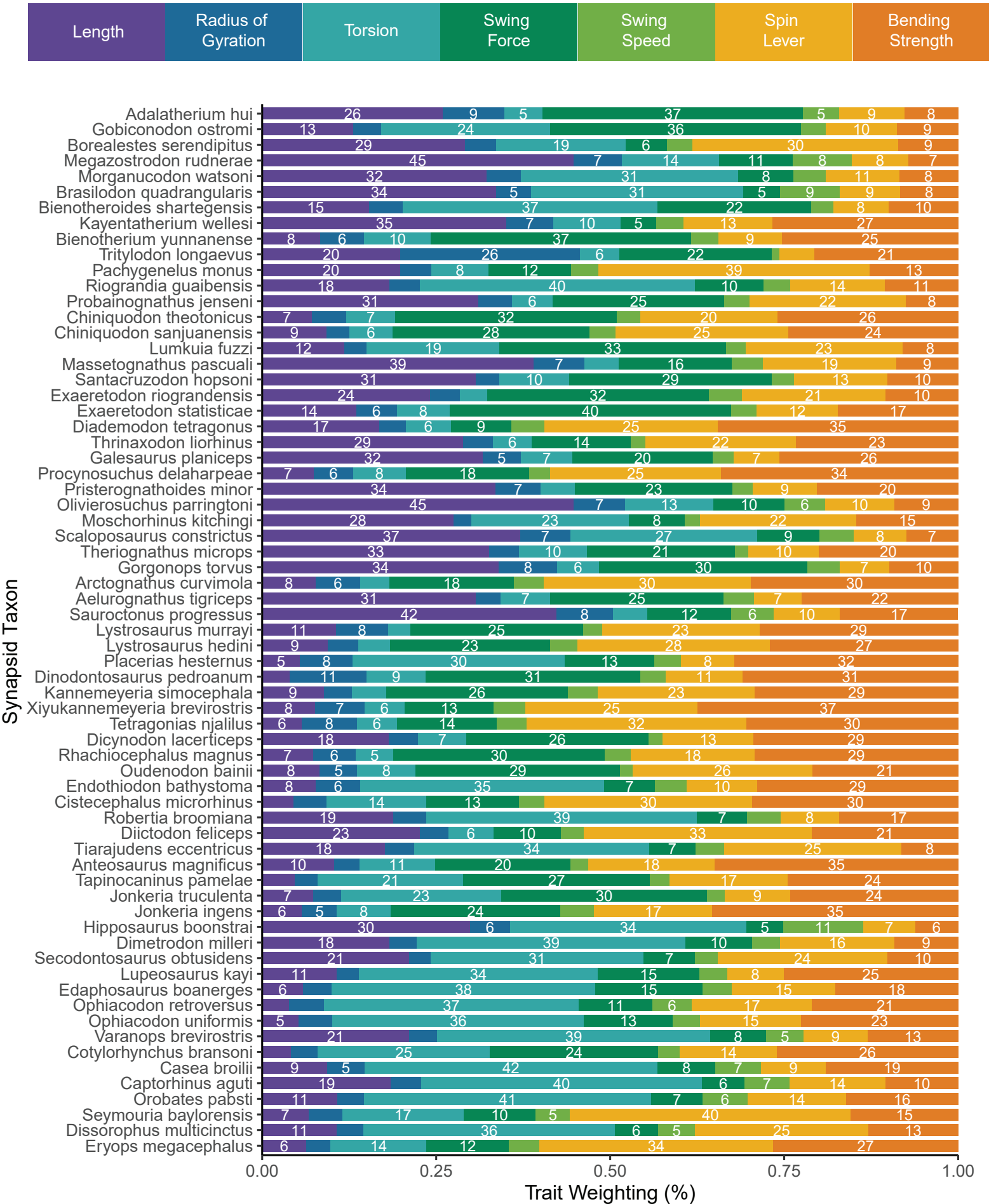

Supplement: S7 Fig — The data underlying this figure can be found in S1 Table and S1 Data. (PDF) [file pbio.3003188.s015.pdf]

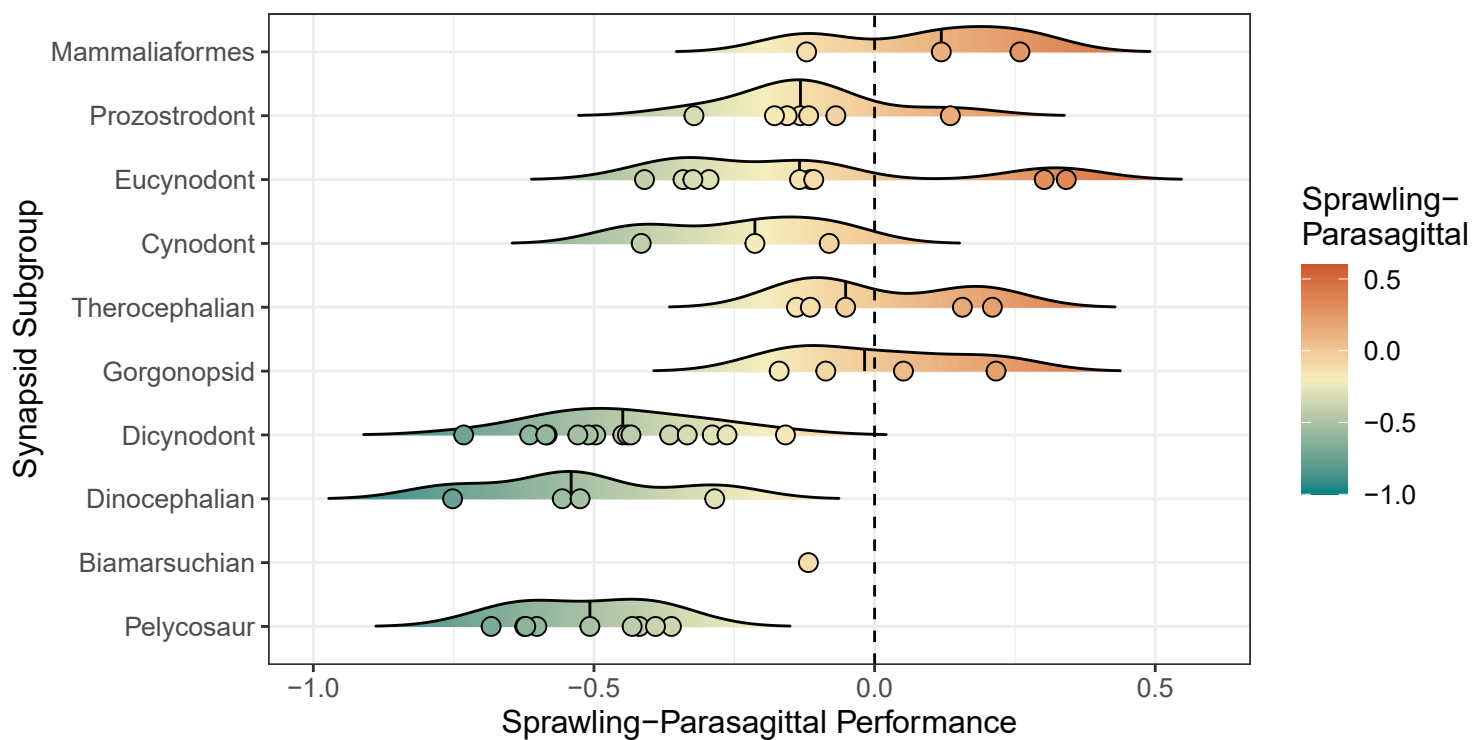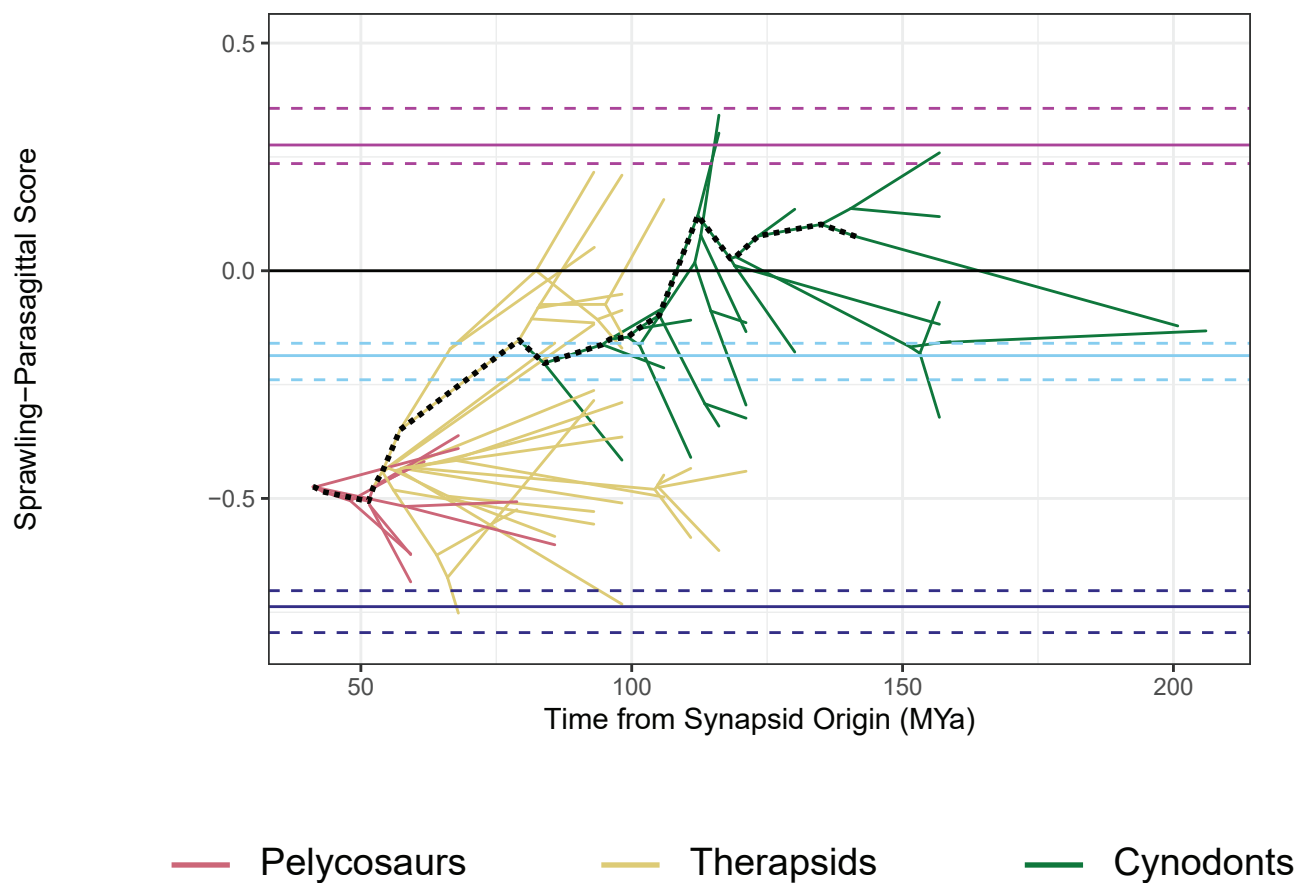

Supplement: S8 Fig — Colored lines on the phenogram show the median, upper, and lower quartile values for extant therians, herptiles, and monotremes. Dashed line on the phenogram indicates the synapsid phylogeny backbone. The data underlying this figure can be found in S1 Table and S1 Data. (PDF) [file pbio.3003188.s016.pdf]

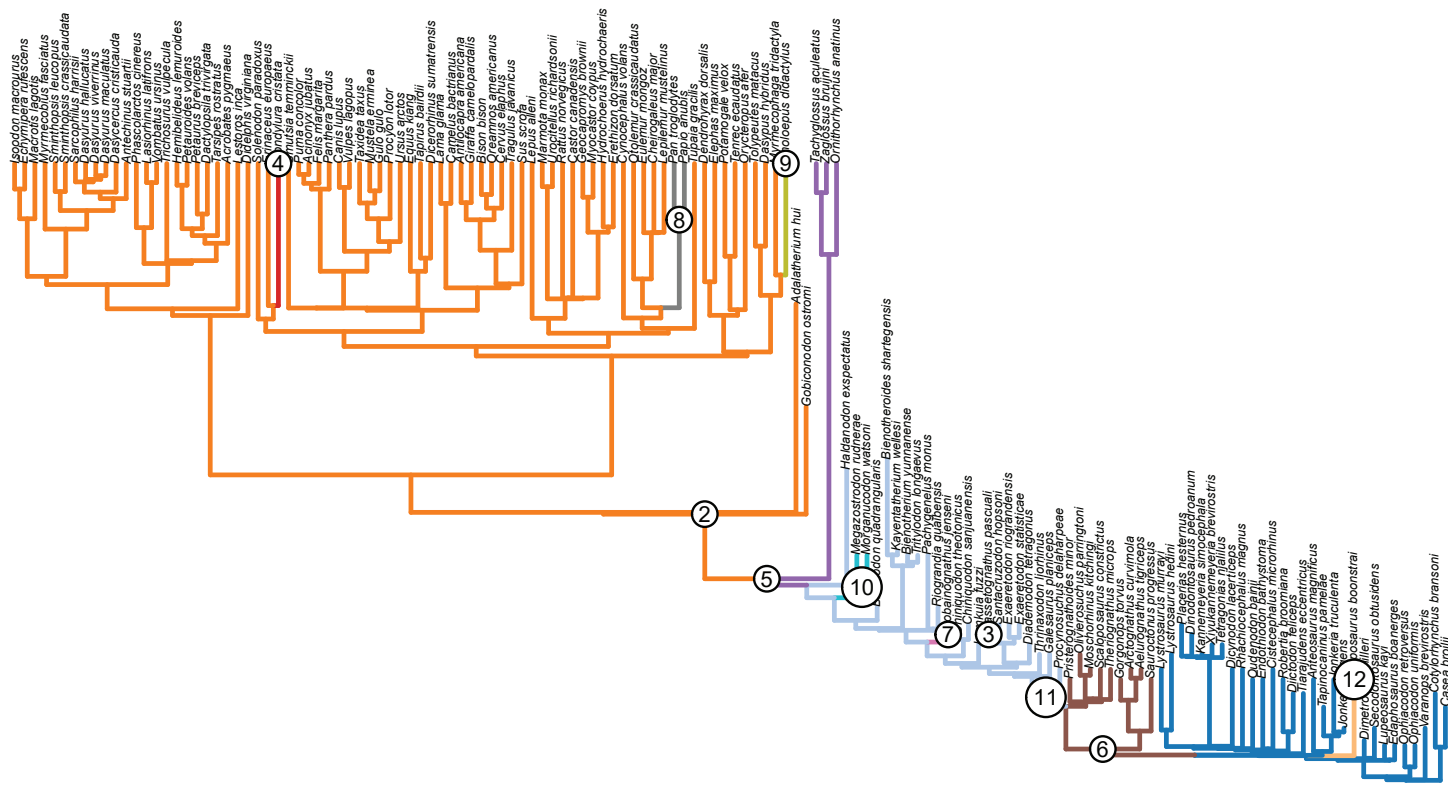

Supplement: S9 Fig — No convergent regimes were detected. The data underlying this figure can be found in S1 Table and S1 Data. (PDF) [file pbio.3003188.s017.pdf]

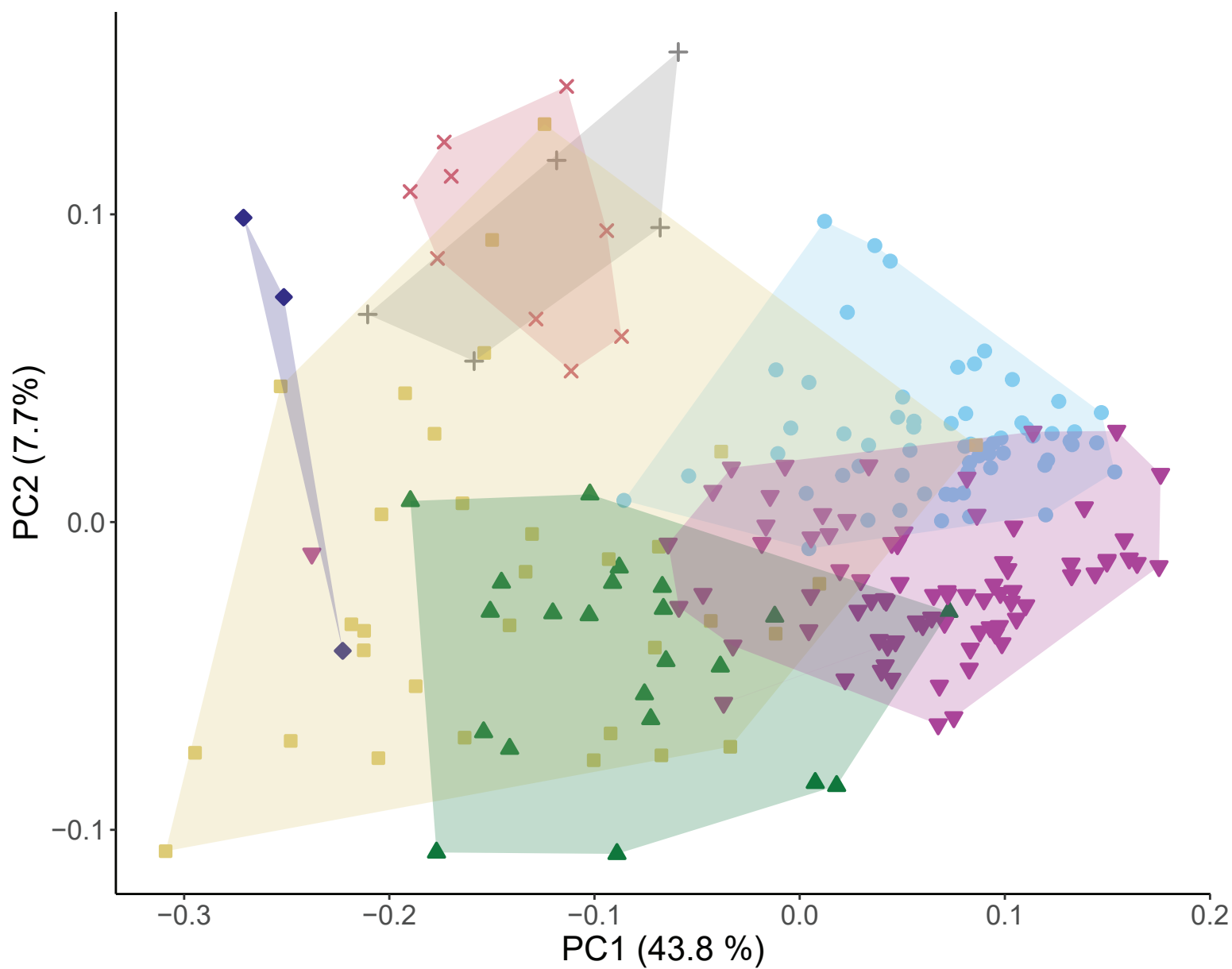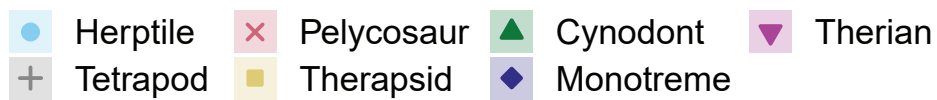

Supplement: S11 Fig — The data underlying this figure can be found in S1 Table and S1 Data. (PDF) [file pbio.3003188.s019.pdf]

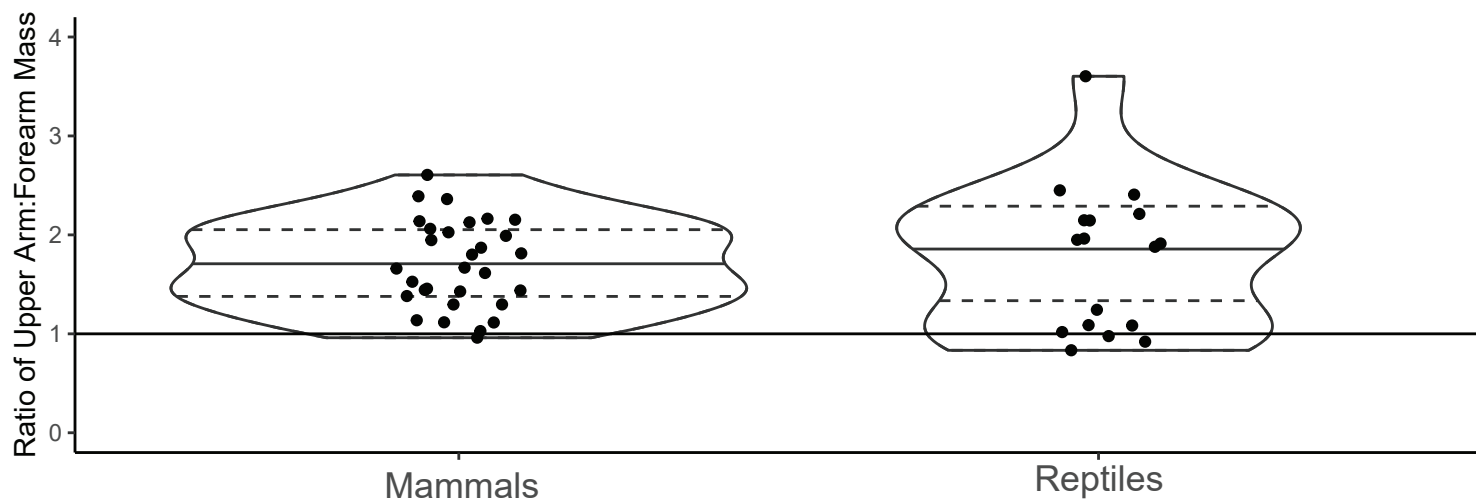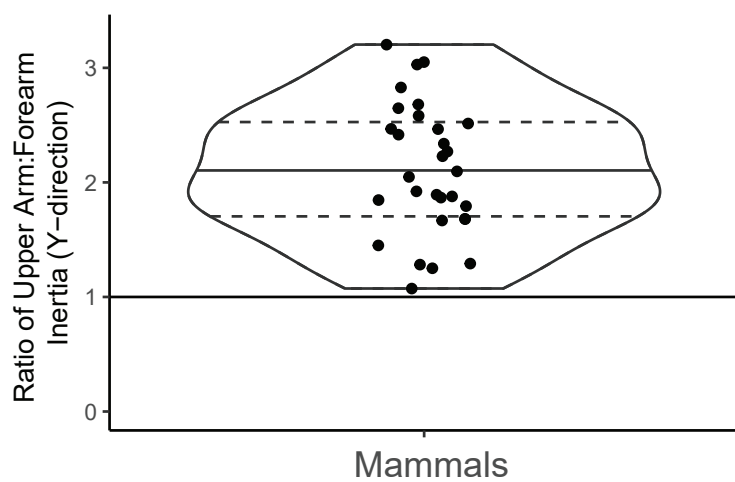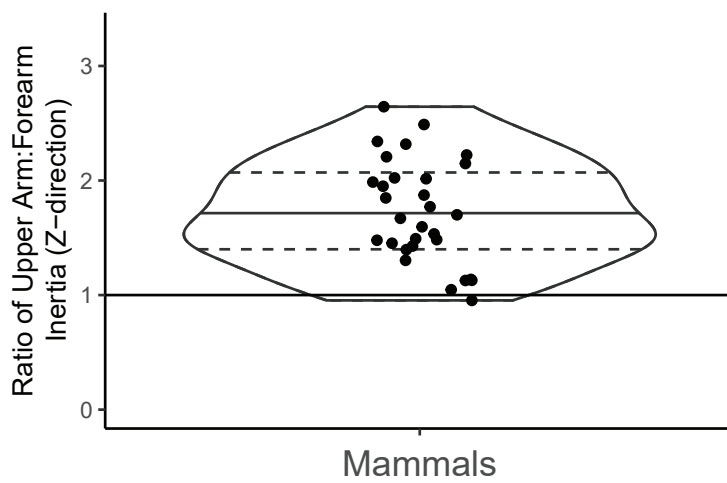

Supplement: S13 Fig — Mammal data from Coatham and colleagues (2021), reptile data from Mcaulay and colleagues (2023). The data underlying this figure can be found in S1 Data. (PDF) [file pbio.3003188.s021.pdf]

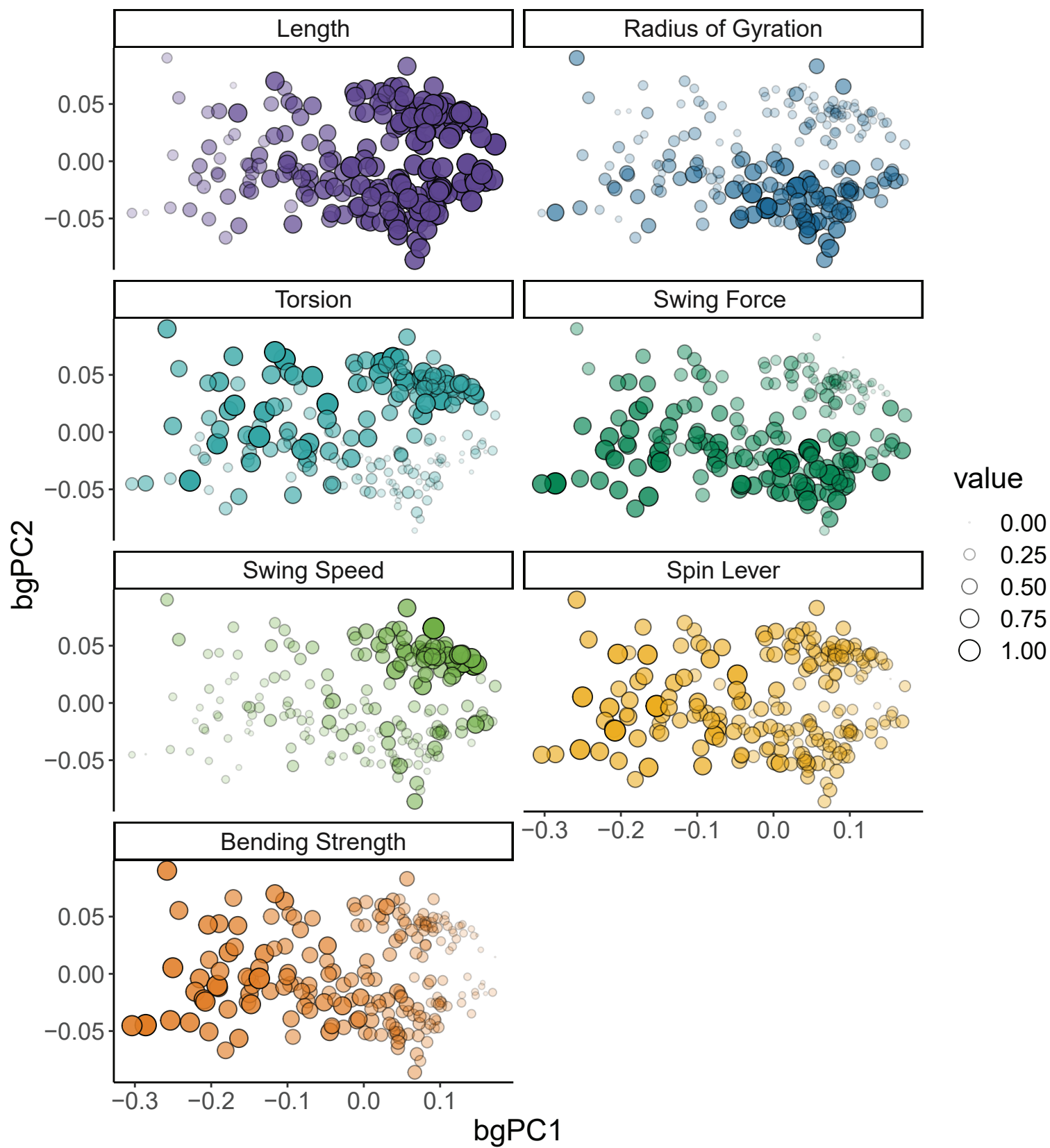

Supplement: S14 Fig — Larger, more opaque points correspond to higher trait values. The data underlying this figure can be found in S1 Data. (PDF) [file pbio.3003188.s022.pdf]

A

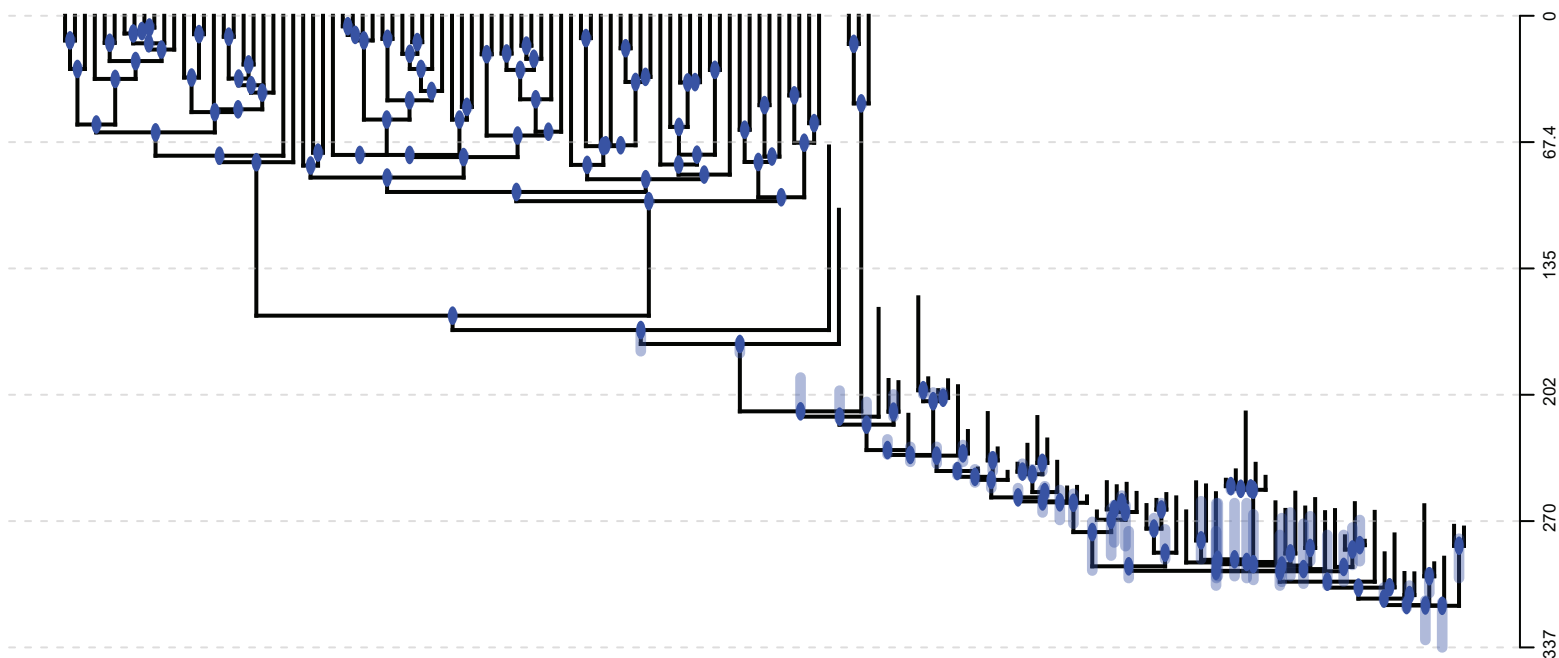

B

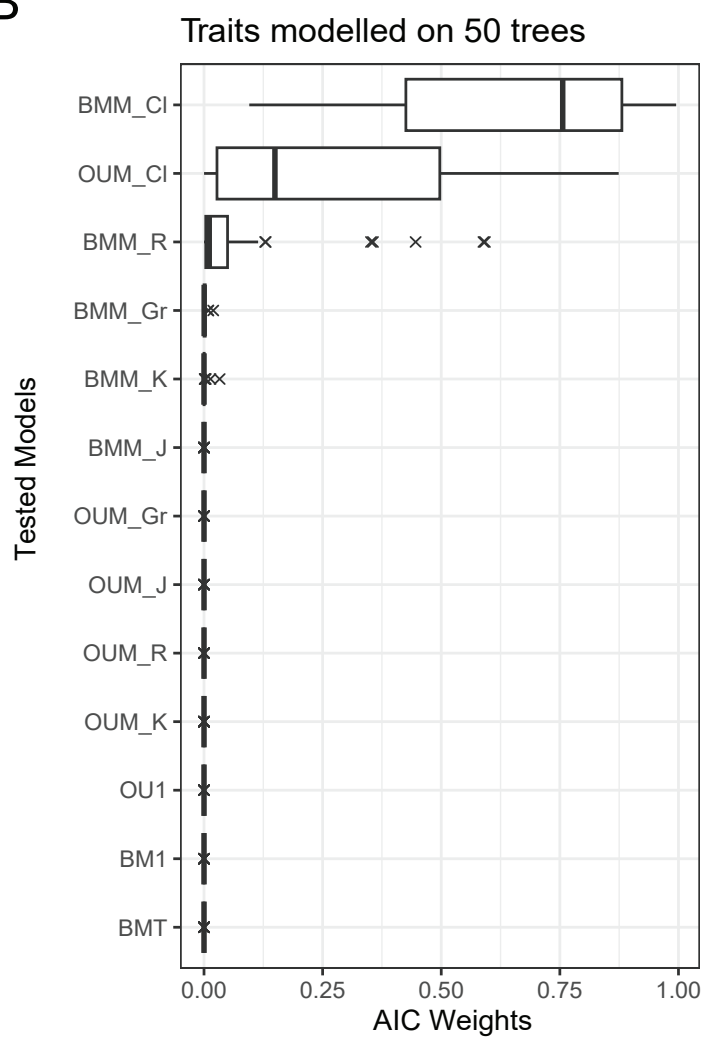

C

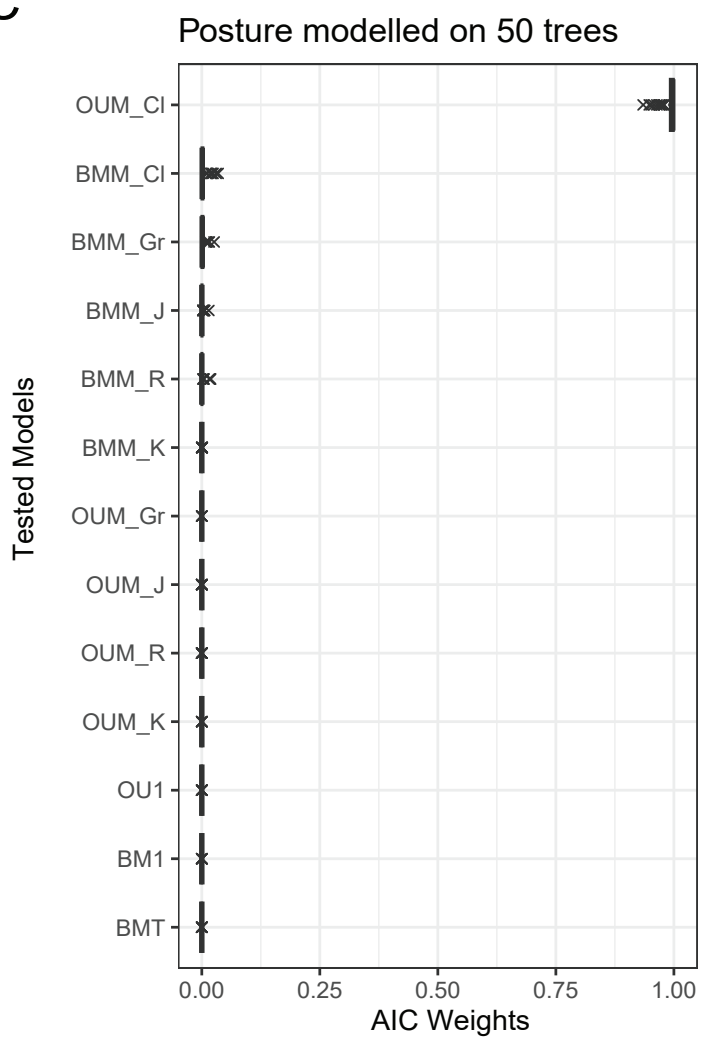

Supplement: S15 Fig — Phylogeny showing ranges of nodal ages across 50 trees (A). AIC weights for each of the models of trait (B) and posture (C) evolution tested using mvMORPH (S5 Fig), across a distribution of 50 trees. Favored models match the preferred models recovered by the main analysis and discussed in the main text (see Results and S4 and S6 Tables). The data underlying this figure can be found in S1 Table and S1 Data. (PDF) [file pbio.3003188.s023.pdf]
