# Supplementary material for: Adaptive landscapes unveil the complex evolutionary path from sprawling to upright forelimb function and posture in mammals
Source: PLoS Biol. 2025 Jun 24;23(6):e3003188. doi: 10.1371/journal.pbio.3003188 (PMC12186895; doi:10.1371/journal.pbio.3003188)
Supplement: S3 Fig — Taxa are listed and numbered in alphabetical order. Taxa are color-coded by group. The data underlying this figure can be found in S1 Table and S1 Data. (PDF) [file pbio.3003188.s011.pdf]

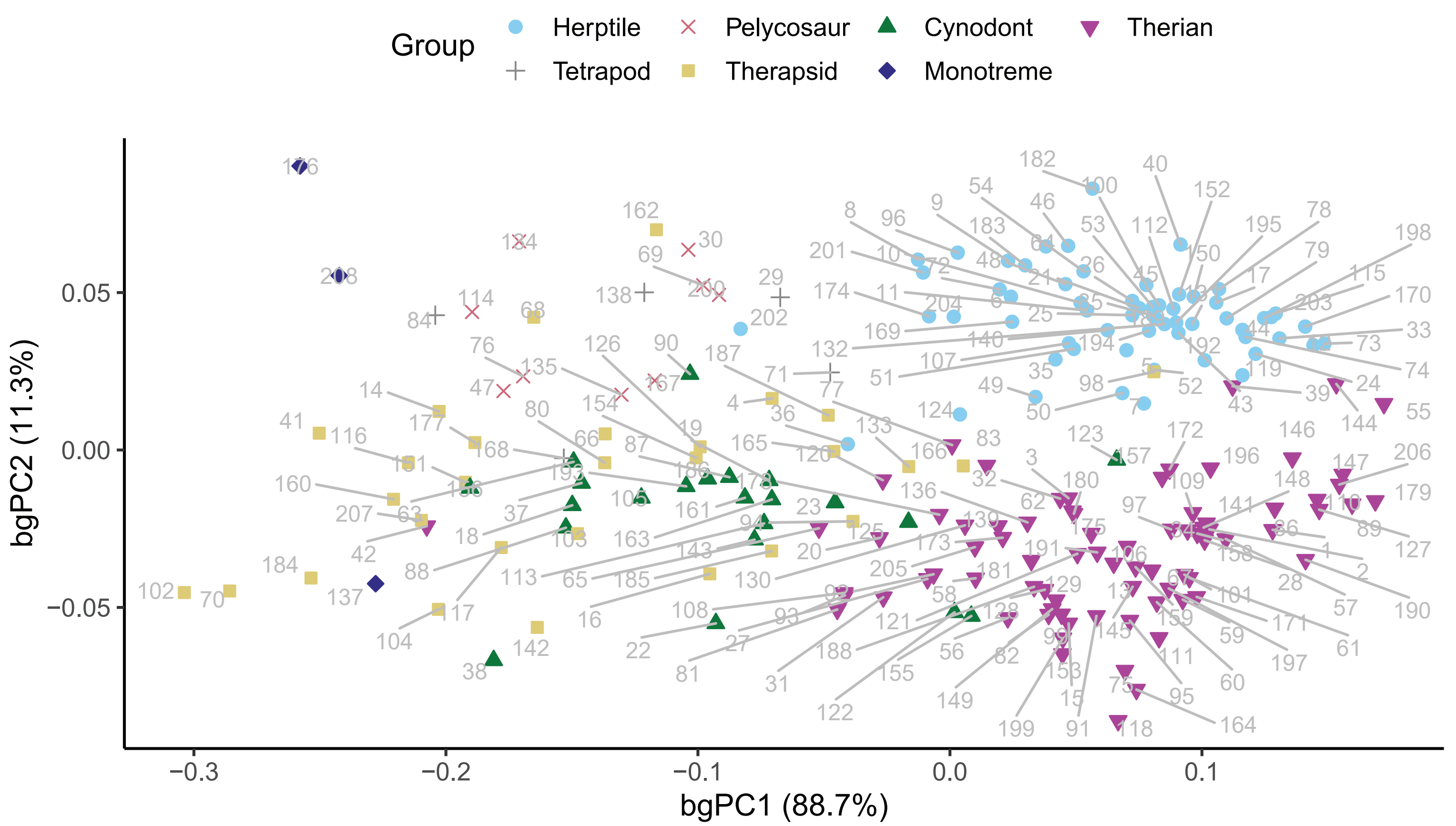

1 Acinonyx jubatus | 2 Acrobates pygmaeus | 3 Adalatherium hui | 4 Aelurognathus tigriceps | 5 Alligator mississippiensis | 6 Alligator sinensis | 7 Alopoglossus copii | 8 Amblyrhynchus cristatus | 9 Ambystoma mexicanum | 10 Ameiva ameiva | 11 Amphiglossus astrolabi | 12 Anolis eugenegrahami | 13 Antechinus stuartii | 14 Anteosaurus magnificus | 15 Antilocapra americana | 16 Arctognathus curvimola | 17 Basiliscus basiliscus | 18 Bienotherium yunnanense | 19 Bienotheroides shartegensis | 20 Bison bison | 21 Bolitoglossa franklini | 22 Borealestes serendipitus | 23 Brasilodon quadrangularis | 24 Brookesia stumpfii | 25 Caiman latirostris | 26 Callopistes maculatus | 27 Camelus sp | 28 Canis lupus | 29 Captorhinus aguti | 30 Casea broilii | 31 Castor canadensis | 32 Cervus elephas | 33 Chamaeleo africanus | 34 Cheirogaleus major | 35 Chelus fimbriata | 36 Chelydra serpentina | 37 Chiniquodon sanjuanensis | 38 Chiniquodon theotonicus | 39 Choloepus didactylus | 40 Chrysemys picta | 41 Cistecephalus microrhinus | 42 Condylura cristata | 43 Cophoscincopus durus | 44 Cophotis ceylanica | 45 Cordylus cordylus | 46 Corucia zebrata | 47 Cotylorhynchus bransoni | 48 Crocodilurus amazonicus | 49 Crocodilus niloticus | 50 Crocodylus johnstoni | 51 Crocodylus porosus | 52 Crocodylus sinensis | 53 Ctenosaura defensor | 54 Cyclura nubila | 55 Cynocephalus volans | 56 Dactylopsila trivirgata | 57 Dasycercus cristicauda | 58 Dasypus hybridus | 59 Dasyurus hallucatus | 60 Dasyurus maculatus | 61 Dasyurus viverrinus | 62 Dendrohyrax dorsalis | 63 Diademodon tetragonus | 64 Dicamptodon ensatus | 65 Dicerorhinus sumatrensis | 66 Dicynodon lacerticeps | 67 Didelphis virginiana | 68 Diictodon feliceps | 69 Dimetrodon milleri | 70 Dinodontosaurus pedroanum | 71 Dissorophus multicinctus | 72 Dracaena guianensis | 73 Draco dussumieri | 74 Draco maculatus | 75 Echymipera rufescens | 76 Edaphosaurus boanerges | 77 Elephas maximus | 78 Elgaria coerulea | 79 Emoia atrocostada | 80 Endothiodon bathystoma | 81 Equus quaggga | 82 Erethizon dorsatum | 83 Erinaceus europas | 84 Eryops megacephalus | 85 Eulamprus quoyii | 86 Eulemur mongoz | 87 Exaeretodon riograndensis | 88 Exaeretodon statisticae | 89 Felis margarita | 90 Galesaurus planiceps | 91 Geocapromys brownii | 92 Giraffa cameleopardis | 93 Gobiconodon ostromi | 94 Gorgonops torvus | 95 Gulo gulo | 96 Heloderma horridum | 97 Hemibelideus lemuroides | 98 Hipposaurus boonstrai | 99 Hydrochoerus hydrochaeris | 100 Iguana iguana | 101 Isoodon macrourus | 102 Jonkeria ingens | 103 Jonkeria truculenta | 104 Kannemeyeria simocephala | 105 Kayentatherium wellesi | 106 Lama glama | 107 Lanthanotus borneensis | 108 Lasiorhinus latifrons | 109 Lepilemur mustelinus | 110 Lepus alleni | 111 Lestoros inca | 112 Lissolepis luctuosa | 113 Lumkuia fuzzi | 114 Lupeosaurus kayi | 115 Lyriocephalus scutatus | 116 Lystrosaurus hedini | 117 Lystrosaurus murrayi | 118 Macrotis lagotis | 119 Madascincus melanopleura | 120 Manis gigantea | 121 Marmota monax | 122 Massetognathus pascuali | 123 Megazostrodon rudnerae | 124 Melanosuchus niger | 125 Morganucodon watsoni | 126 Moschorhinus kitchingi | 127 Mustela erminea | 128 Myocastor coypus | 129 Myrmecobius fasciatus | 130 Myrmecophaga tridactyla | 131 Neusticurus bicarinatus | 132 Notophthalmus viridescens | 133 Olivierosuchus parringtoni | 134 Ophiacodon retroversus | 135 Ophiacodon uniformis | 136 Oreamnos americanus | 137 Ornithorhynchus anatinus | 138 Orobates pabsti | 139 Orycteropus capensis | 140 Osteolaemus tetraspis | 141 Otolemur crassicaudatus | 142 Oudenodon bainii | 143 Pachygenelus monus | 144 Pan troglodytes | 145 Panthera pardus | 146 Papio sp | 147 Petauroides volans | 148 Petaurus breviceps | 149 Phascolarctos cinereus | 150 Phoxophrys nigrilabis | 151 Placerias hesternus | 152 Pogona barbata | 153 Potomogale velox | 154 Pristerognathoides minor | 155 Probainognathus jenseni | 156 Procynosuchus delaharpeae | 157 Procyon lotor | 158 Puma concolor | 159 Rattus norvegicus | 160 Rhachiocephalus magnus | 161 Riograndia guaibensis | 162 Robertia broomiana | 163 Santacruzodon hopsoni | 164 Sarcophilus harrisii | 165 Sauroctonus progressus | 166 Scaloposaurus constrictus | 167 Secodontosaurus obtusidens | 168 Seymouria baylorensis | 169 Shinisaurus crocodilurus | 170 Sitana ponticeriana | 171 Sminthopsis crassicaudata | 172 Sminthopsis leucopus | 173 Solenodon paradoxus | 174 Sphenodon punctatus | 175 Sus scrofa | 176 Tachyglossus aculeatus | 177 Tapinocaninus pamelae | 178 Tapirella bairdii | 179 Tarsipes rostratus | 180 Taxidea taxus | 181 Tenrec ecaudatus | 182 Terrapene carolina | 183 Testudo horsfieldii | 184 Tetragonias njalilus | 185 Theriognathus microps | 186 Thrinaxodon liorhinus | 187 Tiarajudens eccentricus | 188 Tolypeutes matacus | 189 Tomistoma schlegelii | 190 Tragulus javanicus | 191 Trichosurus vulpecula | 192 Triturus cristatus | 193 Tritylodon longaevus | 194 Tropidophorous cocincinensis | 195 Tumbunascincus luteilateralis | 196 Tupaia | 197 Urocitellus richardsonii | 198 Uroplatus fimbriatus | 199 Ursus arctos | 200 Varanops brevirostris | 201 Varanus bengalensis | 202 Varanus komodoensis | 203 Varanus prasinus | 204 Varanus salvator | 205 Vombatus ursinus | 206 Vulpes lagopus | 207 Xiyukannemeyeria brevirostris | 208 Zaglossus bartoni |
