# Supplementary material for: Adaptive landscapes unveil the complex evolutionary path from sprawling to upright forelimb function and posture in mammals
Source: PLoS Biol. 2025 Jun 24;23(6):e3003188. doi: 10.1371/journal.pbio.3003188 (PMC12186895; doi:10.1371/journal.pbio.3003188)
Supplement: S12 Fig — There is a consistent, significant positive relationship between longer humeri and longer forearm elements, demonstrating that humerus morphology encodes useful information about the forelimb as a whole. The data underlying this figure can be found in S1 Data. (PDF) [file pbio.3003188.s020.pdf]

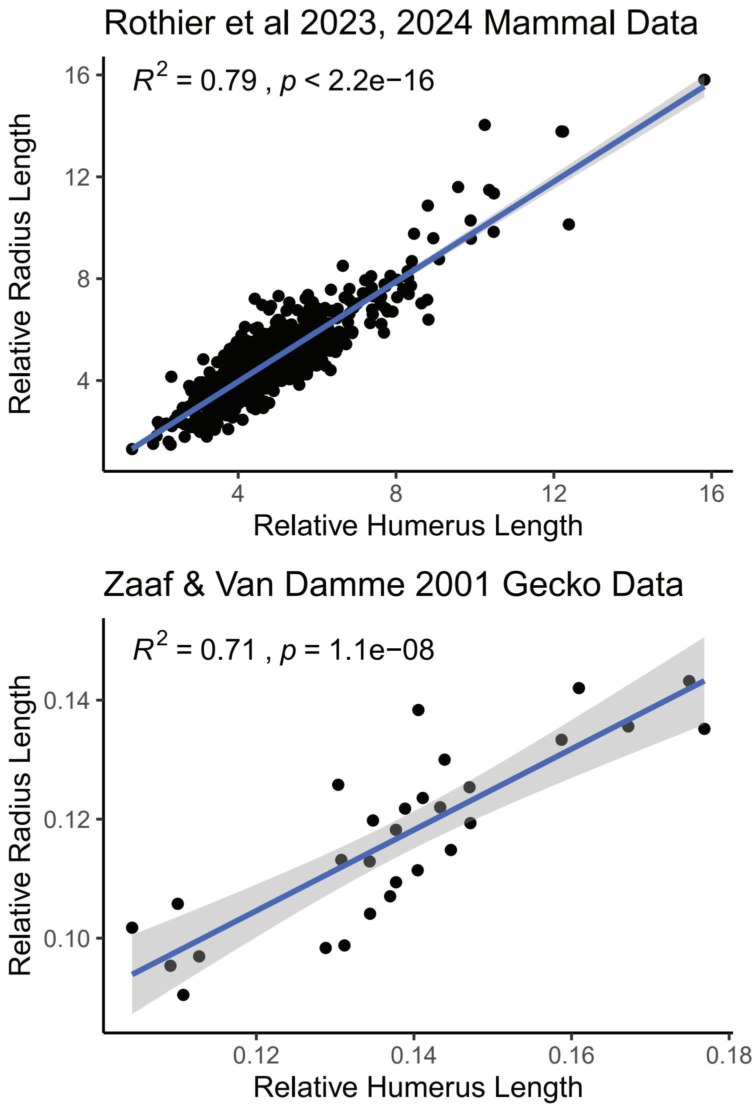

Rothier et al 2023, 2024 Mammal Data
R^2 = 0.79 , p < 2.2e−16
Relative Radius Length
Relative Humerus Length
Iijima et al 2018 Crocodilian Data
R^2 = 0.58 , p = 0.00038
Relative Ulna Length
Relative Humerus Length
Zaaf & Van Damme 2001 Gecko Data
R^2 = 0.71 , p = 1.1e−08
Relative Radius Length
Relative Humerus Length
Christian & Garland 1996 Varanid Data
R^2 = 0.72 , p = 6.7e−07
Relative Radius Length
Relative Humerus Length
