# Supplementary material for: Adaptive landscapes unveil the complex evolutionary path from sprawling to upright forelimb function and posture in mammals
Source: PLoS Biol. 2025 Jun 24;23(6):e3003188. doi: 10.1371/journal.pbio.3003188 (PMC12186895; doi:10.1371/journal.pbio.3003188)
Supplement: S10 Fig — Dashed line on the phenogram shows the backbone of synapsid phylogeny. The data underlying this figure can be found in S1 Table and S1 Data. (PDF) [file pbio.3003188.s018.pdf]

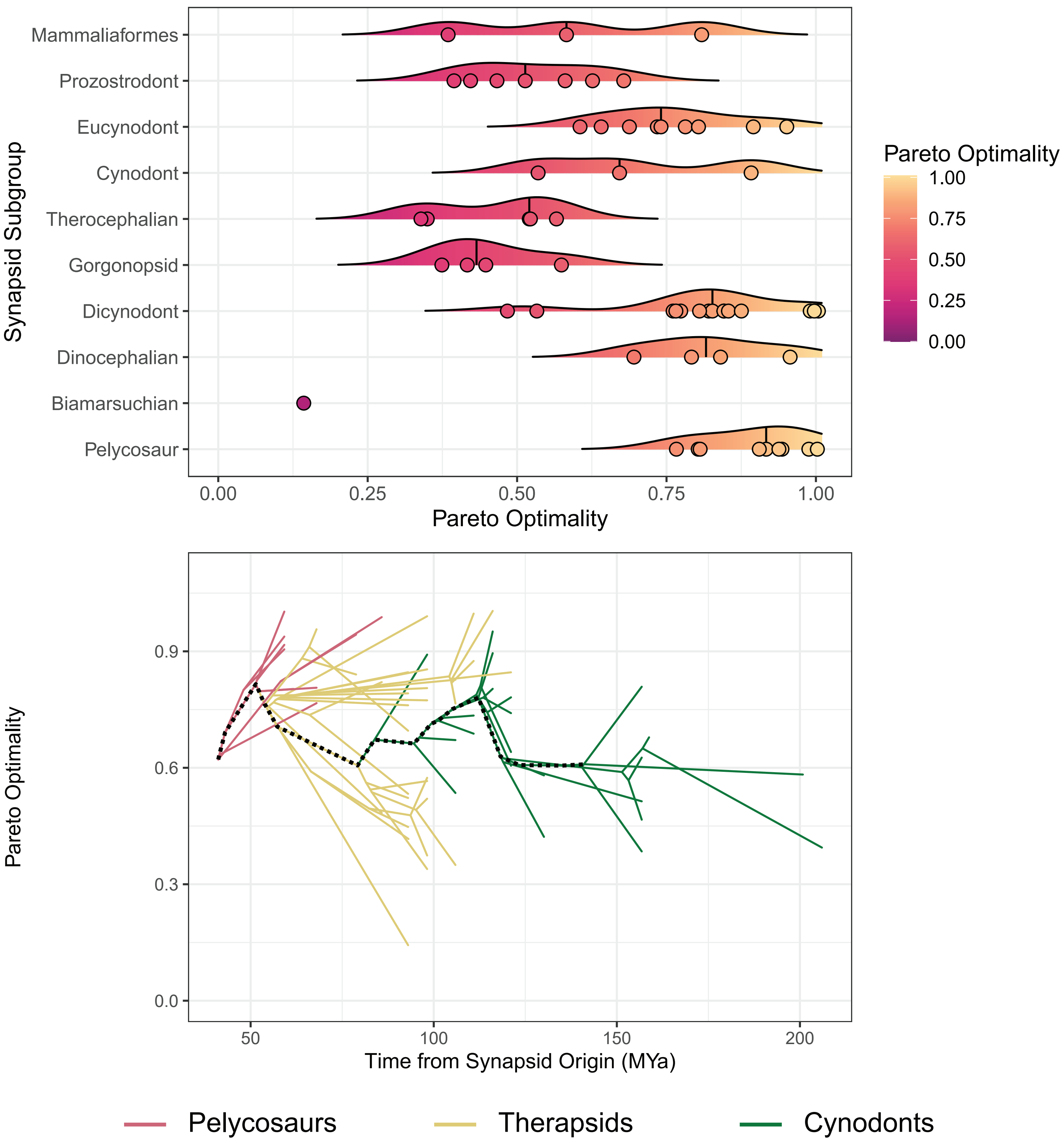

Mammaliaformes
Prozostrodont
Eucynodont
Cynodont
Therocephalian
Gorgonopsid
Dicynodont
Dinocephalian
Biamarsuchian
Pelycosaur
Synapsid Subgroup
0.00
0.25
0.50
0.75
1.00
Pareto Optimality
Pareto Optimality
1.00
0.75
0.50
0.25
0.00
0.9
0.6
0.3
0.0
Pareto Optimality
50
100
150
200
Time from Synapsid Origin (MYa)
Pelycosaurs
Therapsids
Cynodonts
