# Supplementary material for: Adaptive landscapes unveil the complex evolutionary path from sprawling to upright forelimb function and posture in mammals
Source: PLoS Biol. 2025 Jun 24;23(6):e3003188. doi: 10.1371/journal.pbio.3003188 (PMC12186895; doi:10.1371/journal.pbio.3003188)
Supplement: Attachment — Submitted filename: AdaptiveLandscapes_Responses2Reviewers-FINAL.docx [file pbio.3003188.s029.docx]

RESPONSE TO REVIEWS

We would like to thank the four reviewers and the editorial staff for their time in considering our manuscript and providing valuable feedback on our work.

SYNOPSIS:

Our manuscript tackles one of the most profound transitions in vertebrate evolution: **how and when parasagittal posture evolved in the ancestors of mammals**. This question has been discussed for over a century, and the origin of parasagittal posture is considered a key event that led to the ecomorphological radiation of modern mammals. We address this fundamental question in mammal evolution using the most comprehensive (n > 200) 3D dataset yet assembled on fossil synapsid forelimbs and extant comparators. We apply highly quantitative, multivariate approaches incorporating data on limb bone form, function, and evolutionary history to rigorously address postural evolution in synapsids for the first time in decades. Assembling the dataset of synapsid fossil material involved travel across five continents, and provides a holistic, global picture of how the ancestors of mammals transformed their locomotor habits. We incorporated an extensive sample of extant mammals, reptiles and salamanders to validate the inferences drawn regarding our fossil specimens. We devised new homology-free methods for analyzing whole-bone shape variation across our disparate sample and derived novel analytical approaches for integrating disparate concepts of optimality into a unified adaptive landscape framework. Our work provides novel, impactful contributions to the evolution of mammalian posture and limb-bone functional morphology, as well as advancing methodologies for homology-free landmarking and adaptive landscape analyses. Our results show that synapsid forelimb evolution is characterized by successive adaptive radiations and posture was a labile trait that did not fully become parasagittal until the origin of therian mammals.

We address each of the reviewer’s comments in detail below. In summary, after carefully considering the comments of the reviewers and the academic editor, we have made the following major changes to our manuscript:

1. Based on reviewer 1’s comments, we have added additional specimen details to the supplemental information, and additional statistical testing for the effects of size.
2. Based on reviewer 2’s comments, we have performed additional statistical analysis of our adaptive landscape results, and more explicitly link our results to the individual hypotheses outlined in the Introduction. We also added additional tests of explicit evolutionary hypotheses to complement our existing phylogenetic comparative methods. All of these additions support the original contentions of the paper.
3. Based on reviewer 3 and 4’s comments, we have added additional text and supplemental figures clarifying how our functional traits were calculated, as well as their relevance to whole organism performance and justification for their inclusion in our study. We have also revised the treatment of one functional metric (rotational inertia) following comments from reviewer 3.

RESPONSE TO COMMENTS:

Reviewer #1:

This manuscript uses innovative morphometrics and adaptive landscape modelling to interrogate the evolution of the sprawling-parasagittal transition in synapsids. This is clearly a very well written and complete manuscript and I think it should be considered for publication with only very minor revisions.

Thank you for your time reviewing our manuscript and the positive comments about the clarity of our writing.

I have one question about the sampling of fossils. It seems like this semi-automated pseudolandmarking approach would be sensitive to fossil preservation. What was the consideration of fossil specimen selection with regard to preservation/quality? Were any deformed or damaged fossils digitally reconstructed? There are only sparse details about repairing a single specimen in the supplemental table.

Response: All specimens were chosen based on the quality of 3D preservation, with clearly visible morphology at the proximal and distal ends. However, to maximize our sample size, we did include some specimens that required a minimum level of reconstruction, such as fixing cracks or filling holes. We have added additional details of specimen reconstruction to the supplemental information, Table S1.

My only other concern is whether the different optima are body-size correlated. This is discussed briefly on lines 288-293 but I think it could be spelled out a bit more clearly for non-specialist readers. For example, is there a correlation between Sprawling-Parasagittal Score and estimated body mass? What about Pareto Optimality and estimated body mass?

Response: All measurements were corrected for size before further analysis, and in our morphospace large and small animals can be found interspersed (Supplementary Figure 3). We performed additional statistical tests to identify possible relationships between size and shape, functional traits, sprawling-parasagittal posture scores and Pareto optimality, the results of which can be found in the Supplemental Information Tables S2-5.

Our shape and functional trait data do have some relationship with size, but while size is statistically significant, it explains much less variation in our data compared to posture or group membership (S2 and S3 Tables). There is no statistically significant effect of size on our sprawling-parasagittal posture scores or Pareto optimality scores.

Do you want to make a prediction as to what the unique posture/gait of pelycosaurs might have meant for biology/ecology/behaviour(see line 279)- as you do with Therocephalians on line 304-305?

Response: Pelycosaurs are hypothesized to have had a general terrestrial movement ecology, but our data alone cannot go further beyond this interpretation. We reference therocephalians to make the distinction between them and other therapsids, whereas pelycosaurs all have similar, typical morphologies.

Line 362 space needed in "humeriassociated"

Response: This has been corrected.

Reviewer #2:

This is a very clearly written but dense manuscript that seeks to add to our knowledge of the tempo and mode of morphological innovations/changes associated with the so called 'sprawling-parasagittal transition' in vertebrate evolution. Here the researchers focus on the humerus - or rather 3D shape analyses of it.

Response: While our work does investigate when and how the sprawling-parasagittal transition occurred, the terms “tempo and mode” have very specific meanings (referring to rates of evolution and modelling evolutionary processes). We do not use the terms ‘tempo’ and ‘mode’ in our manuscript, and they do not form the primary focus of our paper. In terms of 3D shape analysis, our work goes far beyond only quantifying shape – we incorporate functional trait information along with shape to investigate form-function trade-offs and patterns of evolutionary optimality.

This study makes the point that what was previously known about this issue is gleaned from just one or a few representatives for critical groups (i.e. a very sparse sample of the diversity). This seems to have led previous researchers to propose a stepwise view of postural change. This study increases the sampling somewhat and finds that this stepwise view is not supported in this sample.

Response: While the reviewer has summarized our paper well, we have done far more than increase sampling “somewhat”. Our sample size is over 200 specimens, including 70 3D preserved fossil humeri. This is by far the most comprehensive examination of forelimb evolution across deep time in synapsids, and indeed in tetrapods. Importantly, this sample includes diverse representatives of all of the major clades of non-mammalian synapsids, which allows us to reveal, for the first time, the ways in which the clade explored morphofunctional space over its history. Recognizing the extent to which various synapsid clades experimented with different humerus functions, and the extent of convergent evolution that occurred, is only possible with this type of dense phylogenetic and morphological sampling.

The approach here in terms of shape analyses is not new - they have been used by the authors before in various papers. The shape analyses are not controversial in that there is no doubt that they characterize shape in a way that can be studies comparatively. Although I wonder if such a highly engineered description of  'shape' is necessary or desirable - for example how does selection act on specifically these complex numerical description of shape. This is something that the authors also, even if unknowingly, worry about as they often seek to link their numerical descriptors of shape back to the raw morphology. Anyhow this is a minor point and arguably just a matter of preference rather than any sort of critical problem with what the authors have done.

Response: The reviewer notes that their comment is a matter of preference and not a critical problem. Nevertheless, we would like to address their comment. While shape analyses are not new, the approach we take here replicates the shape of the bones in very high detail, much more so than placing a small set of discrete landmarks on the bone’s surface. Linking numerical descriptors of shape back to the morphologies themselves is not something we “unknowingly worry about”, it is a standard practice in geometric morphometric studies and is entirely appropriate here. Furthermore, we go beyond shape analysis and integrate shape data with functional indices that allow us to explore the selective trade-offs associated with morphological adaptation. Our study makes several major advances on prior work which should be recognized (e.g., an all-new method for automatically landmarking highly disparate anatomical structures, integration of functional and Pareto adaptive landscapes into one study), and we synthesize these insights into a novel narrative of macroevolutionary change in synapsid locomotion. We believe that a real strength of our work is how well we integrate our quantitative results into an understandable, intuitive narrative.

It is true that the authors have increased the sampling from previous work, and this looks numerically impressive. However, when viewed in terms of the sampling across the total number of species in the groups studied is still very space. So, how can we be sure that further sampling will not change our knowledge again?

Response: We do not anticipate any new data to strongly influence the main trends that we find. Mammals cluster with mammals (except monotremes and moles); reptiles cluster with reptiles; and synapsids show a broad range of morphologies across grades and clades. While new data could highlight ecological specialization in different groups, our high-level macroevolutionary conclusions are robust. We assure the reviewer the fossil sample analyzed here is one of a kind, and assembling it required us to travel to 5 continents: Europe, North America, South America, Africa, and Asia. While there are some places we could not visit due to travel restrictions (e.g., Russia), based on our understanding of synapsid anatomy, we do not believe that including these few additional specimens would influence our results here.

I think the manuscript is slightly underwhelming in terms of the testing among the hypotheses proposed and the phylogenetic work is very basic.

Response: It is unclear from this comment whether the reviewer is referring to a basic phylogenetic tree or phylogenetic comparative methods. The academic editor was also confused by this point. With respect to the phylogenetic tree, the goal of this work was not to produce a comprehensive phylogeny of synapsids. This was already achieved in a prior paper (Hellert, Grossnickle et al 2023), which used state-of-the-art metatree analyses to create a total-group synapsid phylogeny including nearly 1900 fossil mammalian and non-mammalian synapsids. We used this most comprehensive tree as the backbone of our study. With respect to phylogenetic comparative methods (PCMs), our paper’s adaptive landscape methods are distinct from PCMs in terms of how they treat adaptive trade-offs and transitions between performance peaks, with adaptive landscapes being the recommended choice for studying continuously varying traits like bone shape and functional trait values (Polly 2020). Adaptive landscapes allow a fully multivariate analysis of traits and interpretations of direct form-function trade-offs that are not possible with PCMs (Polly 2020). However, combining PCMs with adaptive landscapes has the power to further illuminate form-function evolution (Polly et al 2016, Polly 2020, Sanasalone et al 2024), and so we do integrate PCMs into our paper. We use SURFACE to search for shifts in adaptive landscape weighting without constraining them to particular nodes in the phylogeny. However, based on this comment we have now conducted a new set of analyses using mvMORPH to compare explicit hypotheses of landscape evolution. The results of these additional tests favor complex models of posture and trait evolution, with each synapsid grade operating under a distinct evolutionary regime. This contrasts with historical views of synapsid posture evolution as a few “stepwise” changes. Figures illustrating the different hypotheses tested as well as results for best fitting models can now be found in the supplemental information.

Hellert SM, Grossnickle DM, Lloyd GT, Kammerer CF, Angielczyk KD. 2023. Derived faunivores are the forerunners of major synapsid radiations. *Nature Ecology & Evolution*:1–11.

Polly PD. 2020. Functional Tradeoffs Carry Phenotypes Across the Valley of the Shadow of Death. *Integrative and Comparative Biology* 60:1268–1282. DOI: [10.1093/icb/icaa092](https://doi.org/10.1093/icb/icaa092).

Polly PD, Stayton CT, Dumont ER, Pierce SE, Rayfield EJ, Angielczyk KD. 2016. Combining geometric morphometrics and finite element analysis with evolutionary modeling: towards a synthesis. *Journal of Vertebrate Paleontology* 0:e1111225. DOI: [10.1080/02724634.2016.1111225](https://doi.org/10.1080/02724634.2016.1111225).

Sansalone G, Wroe S, Coates G, Attard MRG, Fruciano C. 2024. Unexpectedly uneven distribution of functional trade-offs explains cranial morphological diversity in carnivores. *Nature Communications* 15:3275. DOI: [10.1038/s41467-024-47620-x](https://doi.org/10.1038/s41467-024-47620-x).

The results are largely descriptive and there are no real statistical tests among the hypotheses. Thus, there is only really narrative evidence for one hypothesis over the others. I think a better statistical formulation of the expectations of the hypotheses might be needed to truly show some decisive evidence.

Response: We outline three explicit hypotheses in the introduction of the paper, and throughout the results section we have added new statistical tests to make it clear how these hypotheses are supported or refuted by the results of our manuscript. The new tests include Procrustes ANOVAs for shape data, MANOVAs for trait weight data, and standard ANOVAs for postural landscape scores and Pareto optimality. Results of these statistical tests support our previous “narrative” interpretations and do not change the take-home messages of the paper.

I don't mean to be too critical of this work, I think it is well done and clearly written. However, I think there are limits to what can be said from these data and analytical approaches. I think this work does represent an increase in our knowledge in the area, but I think it is incremental rather than transformative. With this then, and the necessary density of the writing needed to explain the study, I think this manuscript would be far more suitable for a more specialized journal where I think the readership will appreciate the contribution better.

Response: Although we respect the reviewer’s opinion, we politely disagree that our study is “incremental” and that the writing, which explains our results within the context of 325 million years of synapsid evolution, is not of interest to a broad readership. As mentioned above, this is the first-of-its-kind, large-scale 3D analysis of form-function in the limb skeleton of synapsids, using the most up-to-date analytical tools, which our lab has pioneered. We use our unique dataset and multivariate approach to address one of the most profound questions in mammalian evolution, how upright limb posture evolved from our sprawling ancestors.

Reviewer #3:

I have now read the manuscript entitled 'Adaptive landscapes unveil the complex evolutionary path to mammalian forelimb function and posture' submitted to PLOS Biology. In the manuscript the authors use analyses of humerus shape in an extensive sample of fossil taxa to infer function and posture. They then use these data to establish adaptive landscapes and then infer pareto optimality criteria to test the idea that synapids followed an 'optimal' path to the erect parasagittal gate in extant therians. Although there are many thing I really like about this paper including the sample of extinct taxa and the general approach which I found original and exciting, there are also a number of elements that made me question the inferences drawn from the data. I think the overall conclusions make sense and are sound but I wonder whether the data actually support these conclusions. I also found that the authors at time are a bit overly eager to replace shape (what they studied) with function which unfortunately remains unmeasured and only inferred.

Thank you for your time and comments. We appreciate the reviewer’s concerns with respect to function, and we address them below.

Below I detail what I mean by this. I hope my comments and suggestions are useful to the authors and apologise if I have missed information in the manuscript or supplementary information that could have changed my evaluation of the manuscript.

1) sample

Although the authors have an amazing and beautiful sample of extinct taxa I found the sample of extant taxa rather limited and potentially biased. For example, extant monotremes are heavily used as a proxy for the ancestor of early mammals but how relevant are they really? In the discussion the authors themselves highlight this and point out that they are in reality highly derived forms.

Response: There seems to be a misunderstanding of our sampling and our comparison. Irrespective of the number of extant taxa in our study, all reptiles cluster with reptiles and all mammals cluster with mammals, except the sprawling montoremes and moles. We do not believe adding more reptiles or mammals will fundamentally change our morphospace. We also disagree that our sample is biased. There are only three monotremes in our study, compared to 77 therian mammals, and given their phylogenetic position, monotremes are a critical aspect of mammalian taxonomic, morphological, and functional diversity to include in a study about synapsid postural evolution. Further, we never used monotremes as a “proxy” for early mammals. We explicitly note that monotremes seem to have diverged from a somewhat more mammal-like ancestral morphology and moved back into an ancestral synapsid morphofunctional space secondarily. Where our analysis shows functional or morphological similarity between extinct synapsids and monotremes we discuss this as an example of convergent evolution. Animals can be functionally and morphologically convergent and, in fact, convergence is a powerful tool to understand phenotypic evolution. (Note, from a phylogenetic standpoint, extant monotremes are no more derived than extant therians).

2) function

The authors talk about function, performance and posture throughout the manuscript but never do they actually measure these things. All they do is infer proxies thereof based on analyses of shape and morphology. Thus I would strongly urge the authors to be precise in the introduction, results and the discussion of the manuscript and use appropriate language. You did not measure any of these but only used proxies thereof. Moreover, I have some doubts about the relevance of these functional proxies:

Response: The reviewer is correct that we do not directly measure function, and instead infer function from morphological traits or proxies. This is a necessary part of any study involving the fossil record, as direct measurements of functional performance on extinct species are impossible. We have clarified the language in the manuscript to instead refer to “functional traits” or “proxies”. Regarding relevance, all functional proxies used here are derived from previous studies of locomotor evolution in the fossil record and/or are frequently discussed in the biomechanics literature. All our functional proxies are directly related to locomotor performance, and we include additional information about these relationships in the revised version of the Methods.

limb length: you state that humerus is an important contributor to limb length, yet I doubt that it contributes to functional limb length in sprawling animals. In these animals the humerus is oriented more in the horizontal plane and as such does not really contribute to functional limb length. As posture changes humerus length will contribute more to functional limb length but the problem is you infer posture partly from length making this rather circular.

Response: We define functional forelimb length as the distance from shoulder to the hand. In sprawling animals, the motion of the forelimb occurs within the horizontal plane. A longer humerus oriented in this horizontal plane will absolutely increase stride length and so forms a major contributor to the functional length of the limb in sprawlers (Bauwens et al., 1995; Bonine & Garland Jr, 1999; Russell & Bels, 2001; Zamora-Camacho et al., 2014).

Also, we do not infer posture from length – we use length to make inferences about locomotion, but we are quite clear that length does not directly correlate with posture. A good example of this fact is our observation that both therian mammals and modern lizards have both lengthened their humeri relative to those of early tetrapods despite their disparate postures.

Bauwens D, Garland T, Castilla AM, Van Damme R. 1995. Evolution of Sprint Speed in Lacertid Lizards: Morphological, Physiological and Behavioral Covariation. *Evolution* 49:848–863. DOI: [10.2307/2410408](https://doi.org/10.2307/2410408).

Bonine KE, Garland Jr T. 1999. Sprint performance of phrynosomatid lizards, measured on a high-speed treadmill, correlates with hindlimb length. *Journal of Zoology* 248:255–265. DOI: [10.1111/j.1469-7998.1999.tb01201.x](https://doi.org/10.1111/j.1469-7998.1999.tb01201.x).

Russell AP, Bels V. 2001. Biomechanics and kinematics of limb-based locomotion in lizards: review, synthesis and prospectus. *Comparative Biochemistry and Physiology Part A: Molecular & Integrative Physiology* 131:89–112. DOI: [10.1016/S1095-6433(01)00469-X](https://doi.org/10.1016/S1095-6433(01)00469-X).

Zamora-Camacho FJ, Reguera S, Rubiño-Hispán MV, Moreno-Rueda G. 2014. Effects of Limb Length, Body Mass, Gender, Gravidity, and Elevation on Escape Speed in the Lizard Psammodromus algirus. *Evolutionary Biology* 41:509–517. DOI: [10.1007/s11692-014-9285-4](https://doi.org/10.1007/s11692-014-9285-4).

rotational inertia: using volume is very delicate as a proxy for mass as this is dependent on cortical thickness. I doubt you had information on this parameter but maybe I missed it. If you did include variation in cortical thickness in calculating the rotational inertia then please state this explicitly in the methods.

Response: We have revised the treatment of our rotational inertia metric. In light of the reviewer’s comments, we revisited our calculations of rotational inertia and noticed an error in how we had normalized inertia to centroid size. Re-calculating inertia and appropriately normalizing to centroid size resulted in a very different performance surface (Old vs. New Inertia Measure below). Investigating this difference further, we found it was mainly because scaling to centroid size - standard practice in geometric morphometric analyses of shape - does not conserve other functionally relevant metrics of size such as surface area or volume. For a given centroid size, relatively long gracile humeri at the positive end of bgPC1 have relatively total lower volumes, and hence lower rotational inertia (Volume Normalization below).

Our research group has encountered related issues before e.g., Dickson et al (2020) used finite element analysis (FEA) to measure bone stress as a strength metric. Although their bone models were all scaled to centroid size, additional size corrections were still necessary as FEA stress results are typically standardized to model surface area (Dumont, Grosse & Slater, 2009). Reviewing the literature, we found several papers on comparative inertial properties that normalized inertial properties and masses of segments to values for the whole body or complete limb (Isler et al., 2006; Druelle et al., 2017, 2018), but these data were not available for our fossil sample.

To avoid such issues, as well as those raised by the reviewer in their comments, we have revised our inertia metric and propose to use rotational distance (also called radius of gyration) as our metric for inertial performance (Isler et al., 2006; Kilbourne & Hoffman, 2015; Druelle et al., 2018). The rotational distance is defined as the distance from the center of mass to the center of rotation and is commonly normalized to limb (or segment) length for inter- or intra-specific comparisons to illustrate the proximodistal distribution of mass (Isler et al., 2006; Druelle et al., 2017, 2018). A lower rotational distance corresponds to a more proximal distribution of mass and lower rotational inertia when the limb is swung through a horizontal or vertical arc. Calculating performance surfaces for radius of gyration, we found these closely matched our original (“Old” above) inertia surfaces, and downstream analyses and postural inferences were minimally impacted by this change.

Dickson BV, Clack JA, Smithson TR, Pierce SE. 2021. Functional adaptive landscapes predict terrestrial capacity at the origin of limbs. *Nature* 589:242–245. DOI: [10.1038/s41586-020-2974-5](https://doi.org/10.1038/s41586-020-2974-5).

Dumont ER, Grosse IR, Slater GJ. 2009. Requirements for comparing the performance of finite element models of biological structures. *Journal of Theoretical Biology* 256:96–103. DOI: [10.1016/j.jtbi.2008.08.017](https://doi.org/10.1016/j.jtbi.2008.08.017).

Isler K, Payne RC, Günther MM, Thorpe SKS, Li Y, Savage R, Crompton RH. 2006. Inertial properties of hominoid limb segments. *Journal of Anatomy* 209:201–218. DOI: [10.1111/j.1469-7580.2006.00588.x](https://doi.org/10.1111/j.1469-7580.2006.00588.x).

Kilbourne BM, Hoffman LC. 2015. Energetic benefits and adaptations in mammalian limbs: Scale effects and selective pressures. *Evolution* 69:1546–1559. DOI: [10.1111/evo.12675](https://doi.org/10.1111/evo.12675).

Druelle F, Aerts P, D’Août K, Moulin V, Berillon G. 2017. Segmental morphometrics of the olive baboon (Papio anubis): a longitudinal study from birth to adulthood. *Journal of Anatomy* 230:805–819. DOI: [10.1111/joa.12602](https://doi.org/10.1111/joa.12602).

Druelle F, Schoonaert K, Aerts P, Nauwelaerts S, Stevens JMG, D’Août K. 2018. Segmental morphometrics of bonobos (Pan paniscus): are they really different from chimpanzees (Pan troglodytes)? *Journal of Anatomy* 233:843–853. DOI: [10.1111/joa.12894](https://doi.org/10.1111/joa.12894).

Muscle leverage: I was a bit surprised by the rather broad conclusions derived from using a single muscle proxy, the dectopectoral crest. I would argue that this may be a good proxy for humeral retraction but inferring anything about swing from the position of this crest seems a bit tricky. Moreover, the functionally relevant trait to measure is muscle moment arm. Yet, muscle moment arms change throughout the movement and the movement is unknown... so how relevant/useful is this proxy really?

Response: We believe there is some confusion here due to our terminology. We use the term “swing” not to mean the swing phase of locomotion, but to describe taxa whose limbs move through an arc during stance. This contrasted with taxa that emphasize long-axis rotation or “spin” of the humerus during locomotion. We have added addition text to the “Functional traits” section of the results to avoid this confusion and reemphasize this in the Methods. While it is true that moment arms change throughout the joints motion, our prior musculoskeletal analysis of modern taxa demonstrates clear separation based on differences in moment arm distributions across a joint’s whole range of motion (Brocklehurst et al 2022).

Brocklehurst RJ, Fahn-Lai P, Regnault S, Pierce SE. 2022. Musculoskeletal modeling of sprawling and parasagittal forelimbs provides insight into synapsid postural transition. *iScience* 25:103578. DOI: [10.1016/j.isci.2021.103578](https://doi.org/10.1016/j.isci.2021.103578).

Bending strength: the same limitation here as for rotational inertia, without information on cortical thickness any estimation of bending strength becomes extremely difficult to interpret.

Response: It is true that bending strength is somewhat dependent on cortical thickness. However, our chosen metric of bending strength, second moment of area, is a “ *fourth-power function in which external dimensions have overwhelming effects on mechanical strength*” (Lieberman, in Pearson, 2000). Multiple validation studies have shown a close correlation between cross-sectional properties calculated using only external dimensions – as we did here - and those which include internal detail such as cortical thickness (Davies, Shaw & Stock, 2012; Macintosh et al., 2013; Therrien et al., 2016).

We have added these additional references to the Methods.

Pearson OM. 2000. Activity, Climate, and Postcranial Robusticity. *Current Anthropology*. DOI: [10.1086/317382](https://doi.org/10.1086/317382).

Davies TG, Shaw CN, Stock JT. 2012. A test of a new method and software for the rapid estimation of cross-sectional geometric properties of long bone diaphyses from 3D laser surface scans. *Archaeological and Anthropological Sciences* 4:277–290. DOI: [10.1007/s12520-012-0101-8](https://doi.org/10.1007/s12520-012-0101-8).

Macintosh AA, Davies TG, Ryan TM, Shaw CN, Stock JT. 2013. Periosteal versus true cross-sectional geometry: A comparison along humeral, femoral, and tibial diaphyses. *American Journal of Physical Anthropology* 150:442–452. DOI: [10.1002/ajpa.22218](https://doi.org/10.1002/ajpa.22218).

Therrien F, Quinney A, Tanaka K, Zelenitsky DK. 2016. Accuracy of mandibular force profiles for bite force estimation and feeding behavior reconstruction in extant and extinct carnivorans. *Journal of Experimental Biology* 219:3738–3749. DOI: [10.1242/jeb.143339](https://doi.org/10.1242/jeb.143339).

Overall I was wondering how good these functional proxies really are in estimating function, performance or posture... In reality I would have liked to have see a data set on extant mammals where the authors test the validity of these proxies the way they have been calculated before applying them to fossil taxa. If they could provide this and show that they are in fact decent proxies then I would be convinced by the approach. As it stands, everything that follows (calculation of adaptive landscapes etc... is derived from these proxies and thus the overall interpretation as well. This seems like a major limitation of the study.

Response: We are confused by this comment because our study included a large sample of extant mammals (n = 80), as well as reptiles (n = 56) and salamanders (n = 5) *for the exact purpose* *of validating* the use of these proxies in inferring posture and locomotion. Our different extant groups with known postures show both morphological and functional trait differences that can be directly correlated with locomotor behavior. Moreover, the proxies themselves correlate well with real biomechanical data and have been validated as tools for making functional comparisons across species, as evidenced by our extensive list of references provided above.

minor comments:

line 37: what do we really know about the limb muscles in these extinct forms. Or is this inferred from the comparison between reptiles and mammals ?

Response: In fact, we know a great deal about the evolution of musculature in synapsids. Our group recently published two highly detailed monographs that trace appendicular muscles and osteological correlates from pelycosaurs to therian mammals, with extensive anatomical visualization to support interpretations. We have also just released muscle maps of the hindlimb and will be doing a similar publication of the forelimb in the near future (data already collected).

Bishop PJ, Pierce SE. 2023a. The fossil record of appendicular muscle evolution in Synapsida on the line to mammals: Part I—Forelimb. *The Anatomical Record*:ar.25312. DOI: [10.1002/ar.25312](https://doi.org/10.1002/ar.25312).

Bishop PJ, Pierce SE. 2023b. The fossil record of appendicular muscle evolution in Synapsida on the line to mammals: Part II —Hindlimb. *The Anatomical Record*:ar.25310. DOI: [10.1002/ar.25310](https://doi.org/10.1002/ar.25310).

Bishop PJ, Pierce SE. 2024. Reconstructions of hindlimb musculature in extinct pre-therian synapsids. *Bulletin of the Museum of Comparative Zoology* 163:417–471. DOI: <https://doi.org/10.3099/MCZ82>.

line 47: seems like a word is missing after 'presumed'

Response: Thank you for pointing that out. It now reads “presumed functions”

line 52: I agree the humerus is interesting and may provide interesting insights, without the scapula our ability for inferring posture seems extremely limited. The scapula is the primary articulation between the forelimb and the body, not the humerus. As such I would argue that the scapula would be an even better structure to look at. I understand that humeri are probably better preserved in the fossil record, but be honest about why you chose that structure.

Response: It is true that the humerus preserves much better in the fossil record than other postcranial elements, such as the vertebrae or pectoral girdle. This is heavily implied by the line “studying isolated humeri allows extensive taxonomic sampling”. We have changed the language to clarify this point. However, the other reasons we list for studying the humerus - anchoring the major muscle groups that drive locomotion, and its arc of motion directly impacting limb movements – are still valid. We agree that the scapula would be informative, but the idea that it would be “better” than the humerus is a matter of opinion. Regarding the scapula as the primary articulation between the body and forelimb, we have a good understanding of glenoid shape evolution within synapsids, and we correlate our results with those changes (as well as changes to the elbow) in our discussion. However, the scapula experiences different biomechanical loading compared to the humerus and does not contribute to stride in non-mammalian synapsids or lizards (or at least it does so in a very different way than the scapula of a therian mammal).

line 57: you did not measure functional performance so don't write that. Same on line 70 and further in the manuscript.

Response: We measured functional proxies which are directly relevant to functional performance. We have clarified the point, changing the language from “function” to “functional traits” or “proxies” where appropriate.

line 82: your landmarks are not homology free... you use a procrustes superimposition so your landmarks become at least spatially homologous.

Response: The reviewer is correct that our landmarks are spatially homologous. However, it is generally accepted terminology to refer to landmarks lacking anatomical or biological homology as “homology free”.

line 88: extant monotremes re highly derived so how useful are they in the overall discussion ?

Response: Monotremes represent an important component of synapsid evolution and exhibit a sprawling posture. While it is true they possess certain unique derived characters, our data here and from other papers in our research group (Jones et al., 2021; Bishop & Pierce, 2024) suggests that monotremes share many anatomical features with mammaliaforms, and have convergent similarities with other fossil synapsids. Moreover, given their phylogenetic position, any discussion of synapsid limb evolution would be incomplete without considering monotremes in some detail.

Jones KE, Dickson BV, Angielczyk KD, Pierce SE. 2021. Adaptive landscapes challenge the “lateral-to-sagittal” paradigm for mammalian vertebral evolution. *Current Biology*. DOI: [10.1016/j.cub.2021.02.009](https://doi.org/10.1016/j.cub.2021.02.009).

Bishop PJ, Pierce SE. 2024. Late acquisition of erect hindlimb posture and function in the forerunners of therian mammals. *Science Advances* 10:eadr2722. DOI: [10.1126/sciadv.adr2722](https://doi.org/10.1126/sciadv.adr2722).

line 99: you can only infer posture, what you measured is humerus morphology, be precise in your language.

Response: The wording has been changed and now reads that our data are “consistent with their “basal” phylogenetic position, and a unique sprawling posture”.

line 113: you did not quantify function, you only infer it ! just say function was inferred or estimated.

Response: We quantify functionally relevant traits on the bones and infer function from those traits. The wording has been clarified and now reads as follows:

“Function was inferred by measuring seven osteological proxies — bone length, radius of gyration (an important inertial property), torsion, bending strength, and muscle force and speed leverage — for each humerus in our sample (see Methods and S4 Fig).”

line 141: I would not say salamanders and lizard (your herptiles) are characterized by fast limb movements ... rather they have slow humerus movement. This illustrates that you have to be careful in the interpretation of your functional proxies. Here your limited taxon sampling for extant species may bias the data set as well.

Response: We have clarified the language in the text, but here we refer to “relative speed”. While it is true lizards and salamanders are not generally fast, an individual with a short, stocky humerus (and overall shorter limb), similar to a pelycosaur or other early tetrapod, will not move as quickly as one with longer limbs, as longer limbs are known to increase stride length and running speed in lizards (Bauwens et al., 1995; Bonine & Garland Jr, 1999; Russell & Bels, 2001; Zamora-Camacho et al., 2014).

Bauwens D, Garland T, Castilla AM, Van Damme R. 1995. Evolution of Sprint Speed in Lacertid Lizards: Morphological, Physiological and Behavioral Covariation. *Evolution* 49:848–863. DOI: [10.2307/2410408](https://doi.org/10.2307/2410408).

Bonine KE, Garland Jr T. 1999. Sprint performance of phrynosomatid lizards, measured on a high-speed treadmill, correlates with hindlimb length. *Journal of Zoology* 248:255–265. DOI: [10.1111/j.1469-7998.1999.tb01201.x](https://doi.org/10.1111/j.1469-7998.1999.tb01201.x).

Russell AP, Bels V. 2001. Biomechanics and kinematics of limb-based locomotion in lizards: review, synthesis and prospectus. *Comparative Biochemistry and Physiology Part A: Molecular & Integrative Physiology* 131:89–112. DOI: [10.1016/S1095-6433(01)00469-X](https://doi.org/10.1016/S1095-6433(01)00469-X).

Zamora-Camacho FJ, Reguera S, Rubiño-Hispán MV, Moreno-Rueda G. 2014. Effects of Limb Length, Body Mass, Gender, Gravidity, and Elevation on Escape Speed in the Lizard Psammodromus algirus. *Evolutionary Biology* 41:509–517. DOI: [10.1007/s11692-014-9285-4](https://doi.org/10.1007/s11692-014-9285-4).

Lines 154 and further: I was lacking information on how important size is as a driver of your functional proxies... you come back to this in the discussion while saying that behaviors like burrowing or large size/mass may be important drivers of humerus morphology but I would have like to see these caveats addressed earlier in the manuscript.

Response: We have added additional tests on the effect of size on our data, and the results of these tests have been incorporated into the Results section of the manuscript, and additional results tables added the Supplemental information (S2-5 Tables). While size was found to be a statistically significant driver of humerus shape and functional trait weighting, it explained a relatively small amount of variation and had much smaller effect sizes than taxonomic groups. Size had no significant relationship with either transitional landscape posture scores or Pareto optimality.

Discussion: I found the discussion rather long and not always focused on the important elements. I would have like to see the limitations of the data and the functional and postural inferences discussed in greater detail before embarking upon length discussions on evolutionary transitions.

Response: The Discussion places our results and inferences within the full and proper context of 325 million years of synapsid evolution. This topic requires a good deal of consideration because our dense sampling of fossil and extant taxa has revealed the full complexity of synapsid forelimb evolution for the first time, offering many new insights and novel perspectives on previous hypotheses and evolutionary scenarios. Based on our responses above, we hope we have assuaged the reviewer’s concerns regarding limitations of our data and the functional and postural inference drawn from them.

line 392: rather than more sophisticated biomechanical models I think we first need data on extant taxa to validate your proxies.

Response: We refer the reviewer to the extensive lists of references provided in our responses above explaining the validity of the proxies we used. We also once again reiterate that we included a large sample of extant taxa within our dataset (77 therian mammals, 3 monotremes, 56 reptiles and 5 salamanders) to serve as validation for the fossils. Indeed, extant taxa form the majority of our dataset.

Reviewer #4:

[see also the attached PDF]

Using performance landscapes, the authors explore the evolution of the locomotor system (as inferred by humeral morphology) for non-mammalian synapsids in attempt to document changes in limb posture among mammals and their more ancient kin. This is an interesting study, and I think that it is worthy of publication in Journal of Mammalian Evolution. However, there are some points that need to be addressed prior to publication.

Thank you for your time in providing feedback on our study. We presume you meant our study is worthy of publication in PlosBio and that “Journal of Mammalian Evolution” was a typo.

One of the main concerns about this study is that the authors need to devote more of the discussion to the limitations of their study. Their study is indeed informative, but the humerus is only one component of the locomotor system, or even just forelimb anatomy. Therefore, due to other factors, such as muscle anatomy, the anatomy of other limb bones/segments, etc, could it be possible that the taxa that were found to have locomotor postural differences in this study could have in fact had practically similar locomotor styles? In other words, could there be many-to-one mapping of humeral morphology to a given mode of locomotion or posture? This is not something requires a change of the study design or a reanalysis, but it needs to be specifically addressed in the Discussion.

Response: In fact, we specially mention in the text that there appears to be many-to-one mapping, particularly in cynodonts. We are able to make this interpretation as some species with similar humeral shapes and functional traits have differences in other aspects of their forelimb skeleton, such as differences in glenoid or elbow shape that would constrain certain movements. When analyzing our fossil data, we didn’t just look at the humerus, but contextualized the results based on the full forelimb anatomy of the fossils, details of which are found in our Discussion. When it comes to modern taxa, our primary goal was to find the combination of traits that describe sprawling vs. parasagittal posture only, not to identify similarities or differences in locomotor styles within groups, as such a study would require a different analytical approach.

The authors also need to be clear and consistent in their terminology. For instance, the authors specify there is muscle force leverage, muscle speed leverage, and muscle spin leverage. However, at times throughout the text, the authors simply refer to muscle leverage, and it is not clear which of the three is meant or if somehow all three are meant simultaneously. Also, the names of some variables/parameters seem to change between the text and the figures. The authors need to carefully check this for consistency.

Response: We thank the reviewer for pointing this out. We have done our best to make sure terminology is consistent throughout.

The authors need to more carefully consider their biomechanical parameters, or at the very least how they present their parameters. For instance, a second moment of area has to be measured about a specified axis, which the authors don't mention. Likewise, a rotational inertia is also always about a reference axis, not simply a point. However, if the authors were sure to measure each bone in a fixed orientation, then using a simple point could represent an axis coming 90° out of a plane of orientation. So, they need to specify the bone's orientation. Also, the authors might strongly consider trying to measure a parameter that measures resistance to long-axis torsional loads. The parameter that comes to mind in this respect the polar moment of area. The polar moment of area is ideally applied to circular cross sections, but it might still be somewhat informative for the authors' study, given the role that torsional loads play for sprawling taxa (as indicated by the work of Blob & Biewener).

Response: We would like to point the reviewer to our Methods and Supplementary figure S4 which details how our functional traits were measured. For instance, every single bone in our analysis was oriented to the same coordinate system before measuring any parameter; this means all measurements were standardized to a common “reference” system.

The code used to calculate cross-sectional properties calculated a variety of metrics for bone strength, including second moment of area about both x- and y-axes (Ix and Iy), corresponding to dorsoventral bending and anteroposterior bending, as well as polar moment of inertia (J). While Ix, Iy and J varied across slices of each individual bone, we found whole bone summary measurements of Ix, Iy and J (e.g., whole bone averages or mid-section data) were highly correlated with one another. Therefore, we decided to use Ix, dorsoventral bending resistance, as our strength metric as it seemed the most intuitive. We have clarified this in the Methods section.

Kind of going back to my point about many-to-one mapping, I would say that the authors really do not need to discuss the other limb segments when it comes to two parameters in particular: limb length and rotational inertia. Given that the length and mass proportions can greatly vary among limb segments in relation to locomotor modes (at least in mammals and maybe birds' hindlimbs), the authors should mention whether "optimized" humeral length or rotational inertia, as recovered by their study, is sufficient enough to give some indication of overall the overall forelimb's morphology and function, or could there be some ambiguity based upon how length, mass, and (consequently) inertia varies among limb segments. One thing the authors could try is to *qualitatively* report in the Discussion how, say, radius length or olecranon length varies relative to the humerus among taxa clustering in the humerus-based adaptive landscape. Of course, these bones or all their features might not be known for fossil taxa, but this doesn't have to be done in extreme detail. Just enough to give the reader some insight.

Response: The reviewer is correct that relative limb proportion and segment dimensions can and do vary across taxa, especially in species with certain specialized locomotor modes such as cursorial therians. However, our paper deals with general patterns of limb morphology across terrestrial tetrapods (and above the species level), where humeral length and inertial properties can provide powerful insights into the evolution of the limb as a whole.

With respect to limb length, Rothier et al (2023, 2024) have published a very large dataset of mammalian forelimb bone measurements, and across terrestrial mammals their data shows that after accounting for body size, there is a highly significant positive correlation between humerus length and radius length and between humerus length and overall forelimb length. Several studies on different reptile groups e.g., varanids (Christian and Garland 1996), geckos (Zaaf and Van Damme 2001) and crocodilians (Iijima et al 2018) also show positive correlations between upper arm (humerus) length and forearm (radius and/or ulna) length, even after accounting for body size differences.

Regarding inertial properties, Coatham et al (2021) show that the upper arm is consistently more massive and has greater rotational inertia than the forearm across a wide range of mammal species; MacCaulay et al (2023) show a similar pattern in reptiles in terms of segment mass, but did not calculate segment inertia.

We have added references to these papers into the Discussion, and Methods sections of the manuscript. Data from these papers has been collated into a new figure in the supplemental information, S12 and 13 Figures.

Rothier PS, Fabre A-C, Benson RBJ, Martinez Q, Fabre P-H, Hedrick BP, Herrel A. 2024. Of flippers and wings: The locomotor environment as a driver of the evolution of forelimb morphological diversity in mammals. *Functional Ecology* 38:2231–2246. DOI: [10.1111/1365-2435.14632](https://doi.org/10.1111/1365-2435.14632).

Rothier PS, Fabre A-C, Clavel J, Benson RB, Herrel A. 2023. Mammalian forelimb evolution is driven by uneven proximal-to-distal morphological diversity. *eLife* 12:e81492. DOI: [10.7554/eLife.81492](https://doi.org/10.7554/eLife.81492).

Christian A, Garland Jr T. 1996. Scaling of limb proportions in monitor lizards (Squamata: Varanidae). *Journal of Herpetology*:219–230.

Zaaf A, Van Damme R. 2001. Limb proportions in climbing and ground-dwelling geckos (Lepidosauria, Gekkonidae): a phylogenetically informed analysis. *Zoomorphology* 121:45–53.

Iijima M, Kubo T, Kobayashi Y. 2018. Comparative limb proportions reveal differential locomotor morphofunctions of alligatoroids and crocodyloids. *Royal Society Open Science*. DOI: [10.1098/rsos.171774](https://doi.org/10.1098/rsos.171774).

Coatham SJ, Sellers WI, Püschel TA. 2021. Convex hull estimation of mammalian body segment parameters. *Royal Society Open Science* 8:210836. DOI: [10.1098/rsos.210836](https://doi.org/10.1098/rsos.210836).

Macaulay S, Hoehfurtner T, Cross SRR, Marek RD, Hutchinson JR, Schachner ER, Maher AE, Bates KT. 2023. Decoupling body shape and mass distribution in birds and their dinosaurian ancestors. *Nature Communications* 14:1575. DOI: [10.1038/s41467-023-37317-y](https://doi.org/10.1038/s41467-023-37317-y).

When it comes to weightings of their biomechanical parameters, the authors should also give some indication in the text (not just figures) whether the optimizing of a parameter in the landscape entails high or low values of that parameter. This is because, from biomechanical principles, a short humerus might be "optimum" for a given locomotor mode (e.g., a more cursorial taxon), whereas a long humerus might be "optimum" for others (e.g., a more scansorial taxon). You could say the same about rotational inertia. So, I think it's insufficient to simply say a trait is optimized-it's vague from a biomechanical perspective. Please be more specific about this point in the main text.

Response: The adaptive landscape code as implemented in Morphoscape always optimizes for high values of parameter, which are interpreted as higher performance. Therefore, in cases where low values of a trait would be more optimal, such as inertial properties, we used the inverse of the trait values so that low raw numbers gave the highest performance values for optimization. This is clearly stated in the methods section where relevant e.g., line 511 of the revision “We then took the inverse so that lower rotation distance values corresponded to higher performance.”

The use of the term "herptiles" is for me really jarring. Initially, I didn't mind it, but by the end of the manuscript, somehow it became taxing (this is subjective preference, I wholly admit). I think the authors are better off defining (for the purpose of their study) that "reptiles" is taken to mean "non-avian reptiles." As I said though, this point is wholly subjective and my preference.

Response: We use the term herptiles deliberately to refer to both non-avian reptiles and salamanders, not just non-avian reptiles. We found these two groups were often discussed together, and historically the study of reptiles and amphibians has fallen in the field of herpetology, so we used the term herptiles to refer to both. The text was very repetitive if we used “salamander and reptiles” in each instance where they showed similar patterns.
