# Supplementary material for: Adaptive landscapes unveil the complex evolutionary path from sprawling to upright forelimb function and posture in mammals
Source: PLoS Biol. 2025 Jun 24;23(6):e3003188. doi: 10.1371/journal.pbio.3003188 (PMC12186895; doi:10.1371/journal.pbio.3003188)
Supplement: Attachment — Submitted filename: Responses PLOSBio Main 250421.docx [file pbio.3003188.s030.docx]

REVIEWERS' COMMENTS:


Reviewer #2:

At the end of my last referee report, I noted that I find this work interesting and the data collection impressive. Furthermore, from my scrutiny, I believe the methods used in the manuscript have been applied correctly.

However, I would like to emphasize that my role as a referee is to provide my opinion based on the journal's criteria for publication. In this case, the authors do not appear to have taken my previous comments very seriously. Instead, their response comes across as quite combative. In light of their revision and response, I will restate my key points below:

Response: We took the reviewer’s comments very seriously, which is why we felt the need to robustly defend our work. We also made a concerted effort to revise our manuscript, including adding further tests using phylogenetic comparative methods and statistical comparison.

1. Apologies for my sloppy use of "tempo and mode."
I appreciate the clarification provided by the authors on this point.

Response: We thank the reviewer for their appreciation.

2. Shape vs Function
The authors use a homology-free method to characterize shape. I find this approach very interesting, but its relevance to evolutionary analysis is unclear. Shape and function are not synonymous — shape is the product of function alongside many other (often unknown) factors that trade off with it. The hypotheses tested in this manuscript are explicitly about function. Therefore, it would be far more appropriate to measure homologous functional traits directly, or to generate biomechanical traits that are clearly linked to locomotor changes. As it stands, it feels as though the authors have applied a novel method to capture shape, and then retrofitted the resulting data to test hypotheses about function — a conceptual mismatch that is still unresolved.

Response: Our study explicitly measures functional traits, and these traits are biomechanically relevant and linked to locomotor change (see “Measuring functional traits” in the Methods, and our exchanges with Reviewer 3 who was identified as an expert in vertebrate biomechanics). We then integrate these biomechanical data with the shape data collected from the landmarking protocol to understand how both form and function evolve. The conceptual mismatch of using pure shape to test functional hypotheses mentioned by the reviewer is exactly the kind of thing our study sought to avoid.

3. Sample Size
The increased sample size is an improvement, but the dataset remains sparse relative to the total possible number of species. The authors claim that expanding the sample further would not significantly alter the results — but they provide no justification for this statement. As presented, this is pure speculation.

Response: As already stated, we have produced the most comprehensive dataset possible given the state of the world e.g., working at a US institution we were unable to sample specimens from Russia. Therefore, with very few exceptions, we are confident we included as many appropriately preserved fossils (and extant taxa) as possible, and that our results are therefore sound. There are no known extremes of shape disparity we have missed, and we adequately capture known disparity and variation of the fossil record. We also point to review 5’s comment that taxonomical sampling is a “non-issue” and “ No reasonable addition of specimens could be made to this dataset” .

4. Phylogenetic Tree and Comparative Methods
I appreciate the authors' clarification here, but several issues remain:

The Phylogeny: The authors employ state-of-the-art metatree methods to generate a total-group synapsid phylogeny including nearly 1,900 fossil and non-fossil taxa etc. This is indeed a reasonable approach given the data available, and I acknowledge that the authors are working with difficult material. However, this approach comes with major limitations. Most notably, the tree is presented with a degree of topological certainty that is simply not warranted. There is no real discussion of topological uncertainty, which is a significant omission.

Response: We should clarify we did not generate the metatree for this study; the metatree we used was previously constructed and is now published (Hellert et al., 2023). Topological uncertainty was already discussed at some length in the original publication (especially in the supplement) of the metatree analysis (Hellert et al., 2023). Specifically, the main sources of phylogenetic uncertainty within the metatree analysis were large polytomies in mammaliaforms, which represent only a small subset of our fossil taxa (3 species), and certain clades with controversial topological placements, some of which we do discuss in the manuscript e.g., *Adalatherium* (and other allotherians) in lines 388-390. Our primary results center around differences between major grades of synapsids, and the relevant higher-level relationships have been stable and well-established in the literature of many years.

We have added this short segment to the Methods section (lines 632-634): “ While there may be some uncertainty in the phylogenetic position of individual taxa or certain sub-clades (Hellert et al., 2023), the higher-level relationships of Synapsida are well recognized and established in the literature (Sidor and Hopson, 1998; Brocklehurst et al., 2016; Angielczyk and Kammerer, 2018; Matamales-Andreu et al., 2024), and our analyses mainly focus on these high-level relationships, i.e., differences between grades.”

Branch Lengths and Divergence Dates: These are notoriously difficult to estimate accurately, even with large molecular datasets. In a tree built this way, branch lengths are almost certainly poorly estimated (again, this is a known limitation, not a criticism of the authors' effort). However, this uncertainty could have a major impact on the results from comparative methods. There is no attempt to assess or account for this potential issue in the manuscript.

Response: We have now included an additional sensitivity analysis for the effects of branch length on our phylogenetic comparative methods. We re-ran the mvMORPH models on a distribution of trees but re-running the SURFACE models was deemed impractical given time constraints of the revisions. The results support our original analyses. The following figure and text have been added to the methods and supplemental information (S15 Fig and lines 635-643):

“We assessed the effects of uncertainty in branch length by generating a distribution of 50 trees with variations in time scaling (*timepaleophy* function in *paleotree* with *dateTreatment = "minMax"*) and re-running the mvMORPH analyses to obtain a distribution of model support values. Analyzing posture (scores on the transitional landscape) revealed overwhelming support across all sampled trees for the original preferred model; an OU model with peaks for individual synapsid subclades (S4 Table) (S15 Figure). When analyzing limb function (trait weightings for a species adaptive landscape), we found a similar result to the main analysis (S6 Table); high support for models with regimes for individual synapsid subclades (both multi-rate Brownian motion and multi-peak OU) (S15 Figure). This sensitivity testing suggests that the major results of our comparative analyses are not strongly affected by uncertainties in branch length. “

Comparative Methods: The authors use SURFACE to identify 'adaptive landscapes'. While this method is widely used, I would not describe it as particularly cutting-edge. More importantly, the reliability of any comparative method depends heavily on the quality of both the tree and the data. Given the phylogenetic uncertainties discussed above, the results of the SURFACE analysis could easily be compromised. Simply adding a couple of extra statistical tests does not address this underlying structural problem.

Response: We used SURFACE to find major shifts in trait composition making up the functional adaptive landscapes calculated for each species, effectively forming an adaptive landscape of adaptive landscapes. We have responded to the points about topology and branch lengths above.

Conclusion
In summary, while I appreciate the authors' response and their effort to improve the manuscript, several of my fundamental concerns remain unresolved. I continue to feel that the study applies an interesting shape-analysis technique, but does so in a way that does not align well with the core functional hypotheses. Additionally, the phylogenetic uncertainties are not sufficiently acknowledged, despite their potential to undermine key conclusions.

Response: We measure both shape, using our morphometric analyses, and function, using our biomechanically informed trait data. Therefore, our analysis does provide a means of addressing functional hypotheses. We have addressed the points about phylogenetic uncertainty (and other aspects of the phylogenetic methods) above; while we agree that these methods are imperfect, we demonstrate that potential uncertainties do not change the main results of our landscape analysis. The phylogenetic comparative methods (in our minds) are confirmatory tests that reinforce and support the main results of the paper derived from the form-function adaptive landscape analyses. We do not believe topological uncertainty will have an noticeable impact on our results (as stated above). We have now provided an additional test incorporating branch length uncertainty that supports our original results.


Reviewer #3:
[identifies himself as Anthony Herrel]

The revised version of the manuscript entitled 'Adaptive landscapes unveil the complex evolutionary path to mammalian forelimb function and posture' appears much improved. The authors have done a lot of work on the manuscript and I think it can be published in its current form. I appreciate the detailed replies to the reviewer comments provided by the authors. For some functional proxies I am still not 100% sure that the in vivo data needed to validate hem really exist, but I think the authors make solid arguments for most.  

Response: We really appreciate the reviewer’s comments! We agree that more empirical in vivo biomechanical data would be valuable, but we are grateful the reviewer found our arguments based on existing studies to be “solid”.


Reviewer #5:

Summary of Paper
This paper looks at the evolution of posture in non-mammalian synapsids (NMS) using the morphology of the humerus. The comprehensive dataset of NMS provides valuable new insight into the postural transition from NMS to mammals. I encourage publication, but do recommend some minor revisions.

Comments

I noted that previous reviewers took issue with the taxonomic sample. I would argue this is a non-issue. The taxonomic sample is incredibly comprehensive for NMS and arguably the most impressive aspect of the paper. No reasonable addition of specimens could be made to this dataset.

Response: We are grateful to the reviewer for recognizing our efforts and reinforcing this point.

The language change to "functional traits" when referring to functional proxies clarifies the fact that these are not direct measurements of function, and the references to other papers that test these proxies directly with function adequately address the issue of function not being measured directly

Response: Again, we appreciate acknowledgement of this change and we are happy it improved the clarity of the paper.

The new method for semi-automated landmarking is quite clever approach to structures that have the problem of few landmarks.

The explicit hypotheses laid out at the end of the introduction are well addressed throughout the results and discussion. The connection between the analyses and hypotheses were well explained.

My most significant recommendation would be to move away from kriging when creating the adaptive landscapes. Although kriging makes no a prior assumptions, it tends to overfit and produce unrealistically rugged topologies. In this paper, the landscapes for Radius of Gyration, Torsion, and Spin Lever seem to have particularly convoluted topologies (Figure 2A). Something like the second-order polynomial surface used by Dickson and Pierce (2019) would make more sense.  See Rhoda and Angielczyk, "Multivariate functional adaptive landscapes and how we make them" in Friedman, Matt, and Jeffrey Wilson Mantilla. 12TH NORTH AMERICAN PALEONTOLOGICAL CONVENTION UNIVERSITY OF MICHIGAN 17-21 JUNE 2024. (2024).

Response: We are aware of the work done by Rhoda and Angielczyk. We have been in close correspondence with Daniel Rhoda for some time, and Kenneth Angielczyk (who is Daniel’s advisor) is a co-author on this very manuscript! After the abstract referenced by the reviewer was presented, we (the authors along with Rhoda) have discussed the topic of how to construct performance surfaces at length. While polynomial fitting does have its benefits, there are pros and cons of any interpolation function. For instance, polynomials assume there is one peak for any trait while kriging does not make this assumption. In this study, we allowed for multiple peaks and for many-to-one mapping of form-function. We prefer to keep the current approach for the reasons laid out in the Methods, i.e., the lack of a priori assumptions (Lines 582-584).

Methods section Line 583-584 says adaptive landscapes were calculated for each major taxonomic group of interest, but Line 613 in the methods and 128 - 130 in the results say that adaptive landscapes were produced for each species in the dataset. Language should be clarified.

Response: Landscapes were produced for both individual species and major groups. The language has been clarified to reflect this.

Add explained percentage of variation on the bgPCA axes (Figure 1 and S3)

Response: This has been added to the figures. Pease note that bgPC axes explain variance between groups (sprawling, parasagittal and ‘unknown’ for fossils), and each axis explains a greater proportion of this variance than might be seen on a standard PCA; compare with the percentage of total variance explained by PC1 and PC2 in Figure S11.

Reviewer #6:

I am commenting on this revised manuscript after previous reviewer comments. I have a number of points for the authors to address to ensure justification and clarity of methods and interpretations.

Justification for the use of between-groups principal components analysis: I don't see a good reason to forceable separate taxonomic groups when the objective is to analyse functional morphology. I would say that solid justification for the use of bgPCA should be made here (above defending it against potential artefacts that it may generate).

Response: As we are specifically testing hypotheses of shape change as it relates to functional change and posture, we used the between-groups PCA to incorporate a priori posture information and provide better separation than the standard PCA. The wording has been revised to reflect this (lines 486 – 493). The biggest issue with bgPCA is that it can display group differences where none exist. However, we also performed a regular PCA (results in the Supplement) to ensure we did not enforce false groupings in the bgPCA, and we confirm the two morphospaces are very similar to each other.

Slice-based landmarking method: The landmarking method is described as 'novel' whereas it is essentially identical to that pioneered by Polly (2008, Mammalian Evolutionary Morphology, pp 167-196; Polly and MacLeod 2008, Palaeontologia Electronica, 11: 10A) called 'eigensurface analysis'. Acknowledgements of the history of this approach, and its limitations and strengths, should be made clear to the reader.

Response: Thank you for pointing out the Polly reference – we have added it to the manuscript. While we are familiar with much of Polly’s work, we would like to assure the reviewer we were genuinely unaware of these particular contributions and the similarities between the methods are convergent. We have added the following section to the methods (lines 476-478):

“This slice-based landmarking produces a regular array of landmarks on the bone, describing the entire shape in detail, analogous to other morphometric methods e.g., eigensurface analysis (Polly and MacLeod, 2008). It works best on topologically simple structures with a well-defined long-axis, such as limb bones.”

'Unique mode of sprawling': the Abstract suggests that when a group falls into a separate morphological area, it must also have a separate (or 'unique') functional form, essentially equating differences in morphology to differences in function. However, the authors later acknowledge the potential 'many-to-one' mapping that may mean that these different humeral morphologies may in fact have equivalent sprawling function.

Response: We do discuss many-to-one mapping in the manuscript, in reference to more derived therapsid and cynodont-grade synapsids. Our argument for the ‘unique’ sprawling gait of pelycosaurs is based in part on the analyses presented here, as well as other studies of pelycosaur anatomy, functional morphology and locomotor mechanics that suggest they did not use the same walking kinematic as living species (Hopson, 2015; Brocklehurst et al., 2022; Bishop et al., 2023; Bishop and Pierce, 2024).

COMMENTS FROM THE ACADEMIC EDITOR:

I think they can address the limitations posed by reviewer #2 in text. The only alternative would require a new full tree inference where topological uncertainty would be addressed and explicitly estimated to be later used. At this point I am not sure this is possible or desired given all the work already done here.... That said I think "a real discussion of topological uncertainty, which is a significant omission."(reviewer #2) should be done. I think we can condition the acceptance on them being really explicit on this

Response: We now include a short section on possible uncertainties around the phylogeny, specifically the metatree, and we performed an additional sensitivity analysis to see if and how our results might have been affected by differences in branch length estimates.

Also as pointed out by reviewer #6 :"The landmarking method is described as 'novel' whereas it is essentially identical to that pioneered by Polly (2008, Mammalian Evolutionary Morphology, pp 167-196; Polly and MacLeod 2008, Palaeontologia Electronica, 11: 10A) called 'eigensurface analysis'. Acknowledgements of the history of this approach, and its limitations and strengths, should be made clear to the reader."

Response: We were unaware of the methods described in Polly (2008). We have now included them in the manuscript, along with a short description of the strengths and limitations (lines 476-478)

“This slice-based landmarking produces a regular array of landmarks on the bone, describing the entire shape in detail, analogous to other morphometric methods e.g., eigensurface analysis (Polly and MacLeod, 2008). It works best on topologically simple structures with a well-defined long-axis, such as limb bones.”

I noticed that all (or most of) the reviewers emphasized the need to be more clear about the limitations of the different methods (each one a different aspect....). I wonder if it might be beneficial to include a small section on caveats which could even point out what could be done in the future to better take those into account.

Response: We appreciate the editor’s point, however, we respectfully disagree. We do not believe a dedicated caveats section is warranted, as we describe the possible limitations of each method, as pointed out by the reviewers, at the appropriate points in the Methods section.
